# Synthesis of Pyridines, Quinazolinones and Coumarins in Deep Eutectic Solvents: Principles, Methods and Applications

**DOI:** 10.3390/molecules31091503

**Published:** 2026-04-30

**Authors:** Valentina Bušić, Maja Molnar, Mario Komar, Ivana Tomac, Martin Kondža, Martina Jakovljević Kovač, Mirna Habuda-Stanić, Damir Magdić, Lahorka Budić, Dajana Gašo-Sokač

**Affiliations:** Department of Applied Chemistry and Ecology, Faculty of Food Technology Osijek, Josip Juraj Strossmayer University of Osijek, 31000 Osijek, Croatia; vbusic@ptfos.hr (V.B.); mmolnar@ptfos.hr (M.M.); mario.komar@ptfos.hr (M.K.); ivana.tomac@ptfos.hr (I.T.); martin.kondza@farf.sum.ba (M.K.); martina.jakovljevic@ptfos.hr (M.J.K.); mirna.habuda-stanic@ptfos.hr (M.H.-S.); damir.magdic@ptfos.hr (D.M.); lahorka.budic@ptfos.hr (L.B.)

**Keywords:** deep eutectic solvents, pyridine compounds, quinazolinone, coumarine, sustainable synthesis

## Abstract

The synthesis of heterocyclic compounds such as pyridines, quinazolinones and coumarins is a fundamental area of organic chemistry due to their wide application in the pharmaceutical and chemical industries, agro-industry, and other fields of modern technology. As these compounds are produced in large quantities and have significant industrial importance, the development of sustainable and environmentally friendly synthetic approaches has become a key objective of green chemistry. In this context, this review examines the principles, methods and applications of the sustainable synthesis of pyridines, quinazolinones and coumarins in deep eutectic solvents (DESs), a new class of solvents characterized by low volatility, non-toxicity, ease of preparation and recyclability, often from renewable sources. Special emphasis is placed on synthetic strategies that achieve reaction efficiency while reducing environmental impact, including processes without additional catalysts or with reusable catalysts. The paper provides a comprehensive overview of recent advances and highlights the potential of DESs as a viable alternative to conventional organic solvents in the synthesis of bioactive pyridine, quinazolinone and coumarin compounds.

## 1. Introduction

Over the past decade, the scientific community has increasingly and successfully adopted green chemistry as a guiding framework for more sustainable chemical practice [1]. Green chemistry is defined as the design of chemical products and processes that reduce or eliminate the use and generation of hazardous substances. The formalization of this concept, most notably through the twelve principles proposed and established by Anastas and Warner [2], has incorporated environmental responsibility and sustainability into chemical research and development. Earlier contributions, such as Trost’s principle of atomic economy [3,4,5] and Sheldon’s E-factor [6], have laid a quantitative foundation for assessing the environmental impact of chemical processes and have promoted the adoption of more benign synthetic strategies. The importance of green chemistry is particularly evident in the pharmaceutical and chemical sectors. Although research-stage reactions are usually conducted on a small scale, their cumulative environmental impact across numerous laboratories is significant. Furthermore, the subsequent need to redesign inefficient or hazardous synthetic routes for large-scale production can result in significant delays, increased costs, and additional environmental burdens. Modern demands from society and industry require the development of innovative chemical solutions that meet both economic and environmental criteria. This implies abandoning traditional approaches and adopting strategies aimed at reducing the consumption of materials and energy in chemical processes, limiting or eliminating the emission of harmful substances into the environment, increasing the use of renewable raw materials, and improving the durability and recyclability of products. Such an integrated approach is important for increasing industrial competitiveness. Key scientific challenges include developing new synthetic routes that rely on alternative sources of raw materials or highly selective reaction mechanisms, optimizing reaction conditions and selecting more environmentally friendly solvents, as well as designing chemical compounds with lower toxicity and higher safety. In chemical synthesis, the optimal solution involves balancing environmental, health, safety, and economic goals within a single conceptual framework [7].

In line with the principles of green chemistry, a key guideline is the design and use of safer, more environmentally benign solvents. In the development of green reaction media, the greatest attention has been given to deep eutectic solvents (DESs) and ionic liquids (ILs), which, due to their unique physicochemical properties, are considered promising alternatives to conventional volatile organic solvents. Ionic liquids are characterized by negligible vapour pressure, good thermal stability, and the ability to finely tune their properties through the selection of cations and anions, making them attractive for various organic syntheses, including the preparation of heterocyclic compounds such as pyridines. However, despite these advantages, their broader application is limited by several drawbacks, including high synthesis costs, complex and energy-intensive purification and recycling procedures, as well as a variable and sometimes questionable “green” profile, particularly due to the potential toxicity and poor biodegradability of certain ionic species. In addition, high viscosity and possible moisture sensitivity can negatively affect reaction kinetics and operational simplicity.

Due to these limitations, research interest has increasingly shifted towards deep eutectic solvents, which represent a simpler, cheaper, and often more environmentally friendly alternative. Some authors have reported that deep eutectic solvents (DESs) can be considered a new class of ionic liquids (ILs) [8].

Deep eutectic solvents (DESs) represent a promising class of such solvents, combining low volatility, non-flammability, and the potential to reduce environmental and health hazards. They are mixtures of two or more compounds in a specific molar ratio, usually comprising a hydrogen bond acceptor (HBA) and a hydrogen bond donor (HBD), and can often be obtained from natural sources, as in the case of natural deep eutectic solvents (NADESs). However, the modern concept and term “deep eutectic solvents (DESs)” were first introduced by Andrew P. Abbott and his research team in 2003, when they demonstrated that a mixture of choline chloride (ChCl) and urea in a specific molar ratio forms a eutectic liquid with a melting point significantly lower than that of either component alone [9].

Beyond their initial definition, DESs can be further described in terms of their classification, physicochemical properties, and preparation strategies. In this context, they can be divided into several types depending on the nature of their components, including systems based on quaternary ammonium salts and metal chlorides (Type I); hydrated metal salts (Type II); quaternary ammonium salts combined with HBDs such as amides, carboxylic acids, or polyols (Type III); systems involving metal salts and HBDs (Type IV); and non-ionic systems composed entirely of molecular components (Type V). Among these, Type III, most often based on ChCl, are the most extensively explored in organic synthesis due to their accessibility, low cost, and favourable environmental properties [10,11].

DESs exhibit a wide range of physicochemical properties, including tunable polarity, viscosity, density, ionic conductivity, and pH, which depend strongly on the nature and ratio of their components. In general, they are characterized by low vapour pressure and non-volatility, making them attractive alternatives to conventional volatile organic solvents. At the same time, their relatively high viscosity can influence mass transfer and reaction efficiency, particularly at lower temperatures. These features, together with their often-reported low toxicity and biodegradability, have contributed to the growing interest in DESs as potential green solvent systems, although such claims should be considered in relation to the specific components employed [12,13].

The preparation of DESs is generally straightforward and does not involve a chemical transformation in the classical sense. In most cases, the components are combined in a defined molar ratio and heated under stirring until a homogeneous liquid is formed, although alternative approaches such as grinding solid components, vacuum evaporation from aqueous solutions, and freeze-drying have also been described. In practice, DESs are often prepared in situ and used directly without additional purification, which represents a clear practical advantage [14].

The behaviour of DESs is largely governed by their hydrogen-bonding network, which underlies both their melting point depression and their function as reaction media. Through specific hydrogen bond interactions, DESs can stabilize charged or polar intermediates and facilitate proton transfer processes, thereby influencing reaction pathways [15]. As a result, they often serve not only as solvents but also as catalytic media, particularly in condensation and multicomponent reactions [16]. Furthermore, DESs can often play a dual role as both solvent and catalytic medium, reducing the need for additional reagents. Consequently, recent studies have increasingly favoured the application of eutectic solvents over ionic liquids, particularly in the context of sustainable and green chemistry [17].

To date, many review papers on DESs have been published [10,18]. They have found wide applications in separation processes [19], biodiesel production [20], electrochemistry [21], carbon dioxide absorption [22,23,24], pharmaceuticals [25,26], chemical synthesis, and enzyme activation or biocatalysis [27]. DESs are increasingly utilized in the synthesis of polymers, electrodeposited materials, and nanomaterials, which can be further applied in the development of electroactive polymer-modified electrodes. Their versatility and tunable properties position DESs as promising media for advancing electrochemical technologies, particularly in the design and fabrication of electrochemical sensors, where their future application is expected to be of significant importance [28].

Deep eutectic solvents (DESs) are widely used as green and versatile media in various applications involving macromolecular substances. They are particularly employed for the dissolution, extraction, and modification of biopolymers such as cellulose, lignin, and proteins, due to their strong hydrogen-bonding ability and low toxicity. In addition, DESs act as effective reaction media and catalysts in polymer synthesis and functionalization processes. Their environmental friendliness makes them promising alternatives to conventional organic solvents in sustainable materials science and biorefinery applications [29].

In organic synthesis, DESs have been extensively investigated as alternative solvents in the preparation of heterocyclic compounds, especially where traditional organic solvents are unsuitable or environmentally undesirable. Although their greenness is generally emphasized, not all DESs are completely biodegradable or non-toxic, highlighting the need for a systematic assessment of their environmental impact. This review focuses on the synthesis of pyridines, quinazolinones, and coumarins in DESs, and the role of DESs as catalysts in the synthesis of these compounds.

Pyridines, coumarins, and quinazolinones are important heterocyclic compounds with wide applications in the pharmaceutical industry due to their diverse pharmacological properties [30,31,32]. Their chemical structures enable interactions with various biological targets, making them valuable in the development of numerous therapeutic agents [33]. Pyridines are among the most significant compounds in medicinal chemistry. The pyridine ring is present in many drugs because it improves solubility, stability, and bioavailability of active substances [34].

**Figure 1:**
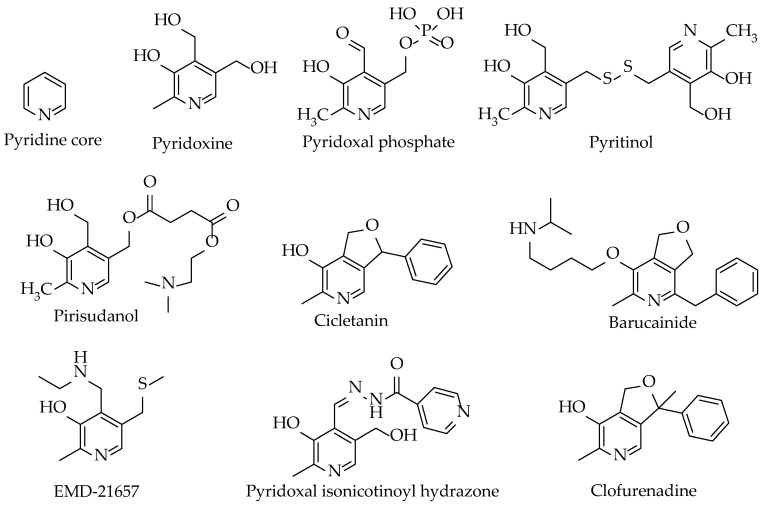
Pyridoxine derivatives approved for use as pharmaceuticals or having reached the stage of clinical trials (CTs).

Pyridoxine-based compounds represent an important class of bioactive molecules, several of which have been approved for clinical use or have reached advanced stages of clinical development (Figure 1). Among the approved drugs, pyridoxine is widely used in the treatment of vitamin B6 deficiency, neurological disorders, sideroblastic anemia, and pyridoxine-dependent epilepsy, as well as in the prevention of isoniazid-induced peripheral neuropathy. Its active coenzyme form, pyridoxal phosphate, is used in conditions associated with hypovitaminosis B6, neurological disorders such as neuritis and neuralgia, and certain cardiovascular and hepatic disorders. Pyritinol, a disulphide derivative of pyridoxine, exhibits neuroprotective properties and is used in the treatment of cognitive disorders, depression, and cerebrovascular diseases. Pirisudanol is used in the treatment of depression, mild cognitive impairment, and fatigue syndrome. Cicletanin is an antihypertensive agent used for both systemic and pulmonary hypertension. In addition to these approved drugs, several pyridoxine derivatives have undergone clinical trials. Pyridoxamine was investigated in phase III trials for diabetic nephropathy, while barucainide, a local anesthetic and antiarrhythmic, also reached phase III before its development was discontinued. Compounds such as EMD-21657 and pyridoxal isonicotinoyl hydrazone have reached phase II for cognitive disorders, i.e., iron overload disease, while clofurenadine has been evaluated in phase I trials for hypertension. These examples highlight the broad therapeutic potential of pyridoxine derivatives in multiple medical fields [34,35].

For example, pyridines are used in the synthesis of antimicrobial [36], anticancer [37], or antihypertensive drugs [9]. The pyridine moiety is also found in vitamin B_3_ (nicotinic acid), which plays an important role in metabolism and is used in the treatment of dyslipidemia [38].

**Figure 2:**
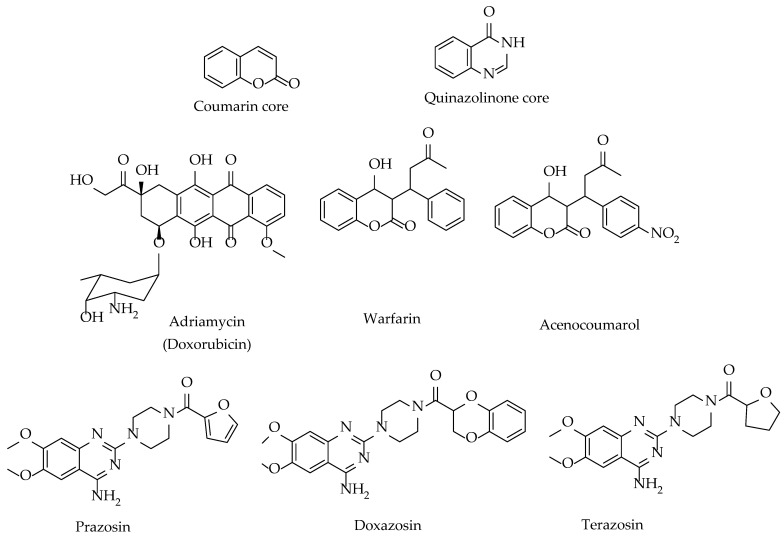
Coumarin and quinazoline derivatives approved for use as pharmaceuticals or having reached the stage of clinical trials (CTs).

Coumarins are well-known for their anticoagulant properties and are widely used in the prevention and treatment of thromboembolic diseases [39]. The best-known example is Warfarin, which acts as a vitamin K antagonist and prevents blood clotting [40]. Besides their anticoagulant effects, coumarins exhibit anti-inflammatory [41], antimicrobial [42], antioxidant [43], and antitumor activities [44]. Due to their ability to interact with enzymes and receptors, coumarins serve as an important foundation for the development of new drugs in cardiovascular and oncological therapy [45]. Among coumarin-based drugs (Figure 2), the most well-known include Adriamycin, warfarin, and acenocoumarol. Adriamycin (doxorubicin) is a potent anthracycline chemotherapy agent used to treat a range of cancers, including breast cancer, lymphomas, sarcomas, and leukemias. Warfarin (Coumadin) is an oral anticoagulant used to prevent and treat thromboembolic events such as deep vein thrombosis, pulmonary embolism, and stroke, particularly in patients with atrial fibrillation, valvular disease, or prosthetic heart valves. Acenocoumarol is a coumarin-derived oral anticoagulant that inhibits vitamin K-dependent clotting factor synthesis and is widely used in Europe as an alternative to warfarin for preventing and treating thromboembolic disorders [46]. Quinazolinones also have significant applications in the pharmaceutical industry, particularly because of their antitumor [47], antibacterial [32], and anti-inflammatory properties [48].

The quinazolinone core is found in many enzyme inhibitors, especially the tyrosine kinase inhibitors used in the treatment of various cancers [49]. These compounds act by blocking the signalling pathways responsible for tumour cell growth and proliferation. Furthermore, some quinazolinones demonstrate antihypertensive [50], anticonvulsant [23], and analgesic effects [51], further expanding their therapeutic potential. Prazosin, doxazosin, and terazosin are quinazoline-derived drugs used in clinical medicine as α1-adrenergic receptor antagonists (Figure 2) [52]. Prazosin is a selective α1-adrenergic receptor antagonist used primarily for hypertension and also for managing post-traumatic stress disorder-related nightmares. Doxazosin is a long-acting α1-adrenergic blocker used in hypertension and benign prostatic hyperplasia, improving blood pressure control and urinary flow. Terazosin is a quinazoline-derived α1-adrenergic receptor antagonist used in the treatment of hypertension and benign prostatic hyperplasia.

Pyridines, coumarins, and quinazolinones play a crucial role in the development of modern medicines due to their diverse biological activities. Their structures enable targeted interactions with biological systems, making them tools in the treatment of numerous diseases, including cardiovascular, infectious, and malignant disorders. Ongoing research on these compounds contributes to the development of new, more effective, and safer therapeutic agents.

For these reasons, it is essential to explore novel and greener approaches for the synthesis of these compounds to align medicinal chemistry with the principles of sustainable development. In recent years, DESs have emerged as promising green alternatives to conventional organic solvents, owing to their favourable physicochemical properties, low toxicity, biodegradability, and ease of preparation. Considering these advantages, investigating the feasibility of synthesizing pyridines, coumarins, and quinazolinones in DESs is an important step towards more environmentally benign and sustainable methodologies. Therefore, this review focuses exclusively on the synthesis of the aforementioned heterocyclic scaffolds in various DES systems, providing a comprehensive overview of existing research and highlighting perspectives that may inspire future advancements in this field.

## 2. Syntheses of Pyridines in DESs

The work of Azizi and Dezfooli [53] describes the use of DESs as “green” and safer alternatives to classical organic solvents in multicomponent reactions. The study demonstrated the efficient and sustainable synthesis of imidazo [1,2-*a*]pyridine via the Groebke multicomponent reaction without catalysts, using ChCl-based solvents. Imidazopyridines are condensed heterocyclic compounds that can be broadly classified as pyridine derivatives. This classification arises from the presence of a conserved pyridine nucleus within the condensed system, their frequent preparation from aminopyridine precursors, and the similar electronic and chemical properties of the pyridine fragment. Despite the additional imidazole ring, the pyridine moiety significantly determines the reactivity and biological properties of these compounds, which justifies their classification as broader pyridine derivatives [54].

As part of the optimization of reaction conditions, different deep eutectic solvents (DESs) were evaluated in a model reaction, all based on choline chloride (ChCl) in a 2:1 molar ratio combined with various hydrogen bond donors or Lewis acids. The DES systems tested included urea:ChCl, SnCl_2_:ChCl, LaCl_3_·6H_2_O:ChCl, glycerol:ChCl, ZnCl_2_:ChCl, and *para*-toluensulfonic acid (PTSA):ChCl. The highest reaction efficiency was achieved with the urea:ChCl system, where a clear increase in yield was observed with rising temperature, from 65% at 25 °C to 90% at 100 °C. The optimal conditions for this system were identified at 80 °C, providing a high and reproducible yield of 87% (87, 85, 85, and 78%), indicating good reproducibility and reaction stability. In comparison, other DESs showed significantly lower efficiencies at 80 °C: ZnCl_2_:ChCl gave a moderate yield of 76%, SnCl_2_:ChCl 60%, LaCl_3_·6H_2_O:ChCl 56%, while glycerol:ChCl was markedly less effective with a yield of 30%. *Para*-toluene sulfonic acid:ChCl did not produce any product (0%), indicating its unsuitability for the investigated reaction. Overall, urea:ChCl proved to be the most efficient eutectic solvent for the model reaction, both in terms of yield and overall reaction performance across a broad temperature range.

**Scheme 1:**
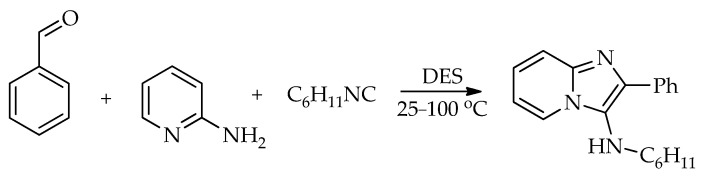
Groebke multicomponent reaction [53].

Six different systems were tested, with the ChCl:urea combination proving to be the most successful, giving good yields (57–87%) with relatively short reaction times (2–6 h) (Scheme 1). Based on the results, it was concluded that the method is environmentally friendly, uses a biodegradable solvent, allows simple product work-up by filtration, and offers general experimental simplicity.

**Scheme 2:**
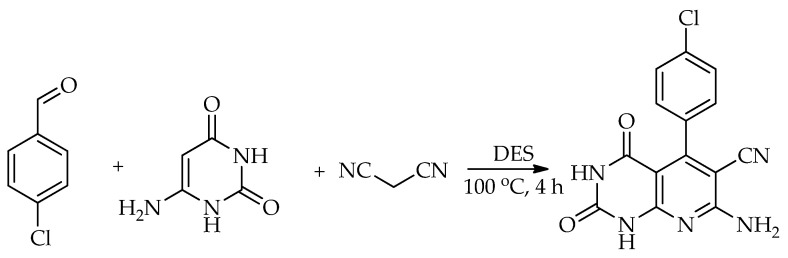
Synthesis of pyrido [2,3-*d*]pyrimidines in DESs [55].

The paper Aryan et al. [55] describes a simple and efficient multicomponent synthesis of novel, highly substituted pyrido [2,3-*d*]pyrimidines in DESs (Scheme 2). Various DESs were prepared, ChCl:urea, ChCl:glycerol, ChCl:lactic acid, glucose:glycerol, lactic acid (Lac):glycine, Lac:glucose, Lac:histidine, and Lac:citric acid, and used as solvents and catalysts. Among those tested, ChCl:urea was found to be the best based on product yield (91%). The reusability of the DES was examined; the DES mixture was successfully recovered and reused three times with only a minimal reduction in activity.

**Scheme 3:**
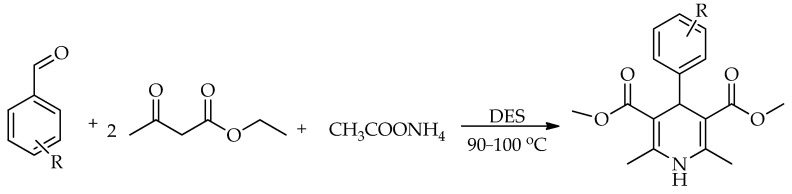
Synthesis of 1,4-dihydropyridines in piperidinium bromide and fluoride-based DESs [56].

*N*-methyl piperidinium bromide and fluoride-based DESs were efficient reaction media for one-pot reactions to synthesize *N*-heterocycles like 1,4-dihydropyridine, acridine, quinoline and indole [56] (Scheme 3). Different hydrogend bond donors were used: urea, methanol and ethanol. The reaction proceeds as a pseudo-four-component reaction (pseudo MCR [57]), allowing one-step synthesis of target compounds from three different starting materials under mild conditions. Pyridines were obtained in high yields (70–81%), with the DES serving both as solvent and catalyst. A comparison of DESs based on bromide and fluoride anions showed that systems with fluorides achieve higher yields. This may be due to the fact that fluoride anion exhibits strong interaction with HBD due to greater electronegativity compared to bromide anion and creates stronger, denser hydrogen-bonding networks.

**Scheme 4:**
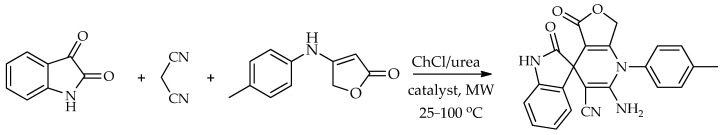
Reaction of isatin, malononitril and 4-(*p*-tolylamino)furan-2(5*H*)-one [58].

The DES based on ChCl:urea proved to be an efficient and environmentally friendly reaction medium for the one-pot, three-component synthesis of spiro-oxindole dihydropyridines under microwave irradiation [58]. The use of this DES system enabled high to excellent yields in the reaction of isatins, malononitrile, and anilinolactones, highlighting its crucial role in enhancing both the efficiency and sustainability of the process (Scheme 4). Within this ChCl:urea medium, a magnetically separable molybdenum catalyst immobilized on Fe_3_O_4_/graphene oxide was applied for the first time in the synthesis of spirooxindole dihydropyridines via a one-pot multicomponent reaction. In this approach, the DES not only serves as a green reaction environment but also contributes to simplified work-up, improved catalytic performance, and overall environmental compatibility.

**Scheme 5:**
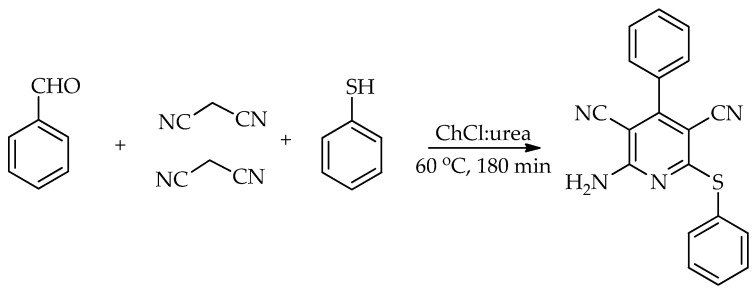
Three-component synthesis of highly substituted pyridines [59].

Azizi et al. [59] describes a one-step, three-component pseudo MCR synthesis of highly substituted pyridines from structurally diverse aldehydes, aromatic thiols, and malononitrile using a ChCl:urea-based DES as a green solvent and catalyst (Scheme 5). The method is cost-effective, environmentally benign, scalable, and provides moderate to good yields (60–82%) in short reaction times (80–240 min), with the added advantage of DES recyclability without loss of activity over at least three cycles. In addition to ChCl:urea, other DESs have been tested. ChCl:glycerol DES achieved good conversion, whereas DESs using malonic acid, ZnCl_2_, SnCl_2_, and *p*-toluenesulfonic acid as HBDs were the least effective reaction media. To determine the role of DESs in promoting the reaction, the starting components of DESs ChCl, urea, and glucose were tested. Low conversion occurred when these substances were used as catalysts and reaction media, further indicating the key role of DESs.

**Scheme 6:**
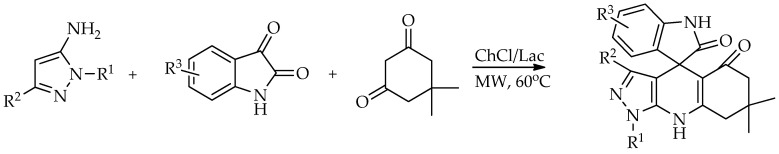
Synthesis of spiro[indoline-3,4′-pyrazolo [3,4-*b*]pyridines] in NADES [60].

This study of Zhang et al. [60] presents a green and efficient one-pot, three-component synthesis of spiro[indoline-3,4′-pyrazolo [3,4-*b*]pyridines] and related spiroheterocycles from 1*H*-pyrazol-5-amine, isatin, and enolizable C–H activated compounds, combining microwave-assisted organic synthesis (MAOS) with a ChCl:Lac-based natural DES (NADES) (Scheme 6). The combination of the DES and microwave synthesis allows the reaction to proceed rapidly, with the desired products obtained within 15–25 min at high yields of approximately 90–95%. This system is environmentally friendly as it uses biodegradable and non-toxic solvents. An additional advantage is that the DES serves both as a solvent and as a catalyst, simplifying the process and eliminating the need for extra chemicals. The method demonstrates good scalability, having been successfully performed on a larger (100 mmol) scale while maintaining high yields, which is important for potential industrial applications.

**Scheme 7:**
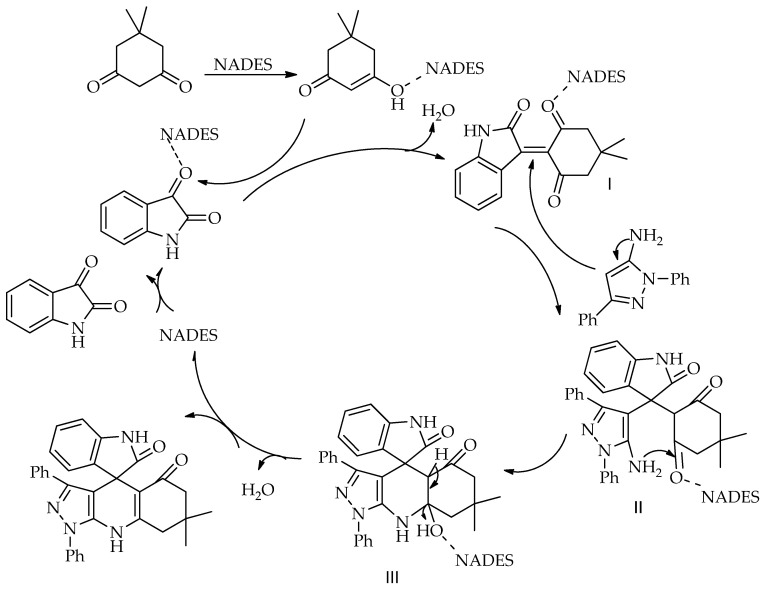
The proposed mechanism for the formation of spiro[indoline-3,4′-pyrazolo [3,4-*b*]pyridines] in the NADES [60].

Based on the literature [61,62] and the obtained results [60], a plausible reaction mechanism for the synthesis of 7′,7′-dimethyl-1′,3′-diphenyl-1′,7′,8′,9′-tetrahydrospiro[indoline-3,4′-pyrazolo [3,4-*b*]quinoline]-2,5′(6′*H*)-dione in a NADES is proposed (Scheme 7) [60]. The first step is a Knoevenagel condensation between isatin and 5,5-dimethylcyclohexane-1,3-dione, forming intermediate I in the presence of the NADES. This is followed by a Michael addition of 1,3-diphenyl-1*H*-pyrazol-5-amine to the C=C bond of intermediate I, resulting in adduct II. Next, an intramolecular cyclization condensation occurs between the amino and carbonyl groups of Michael adduct II, yielding intermediate III. Finally, the elimination of a water molecule from intermediate III affords the target product. The NADES acts as both a solvent and a catalyst through hydrogen bond formation. Its strong interaction with carbonyl groups facilitates the formation of the Knoevenagel condensation product and enhances the electrophilic character of the carbonyl carbons in intermediates I and II.

**Scheme 8:**
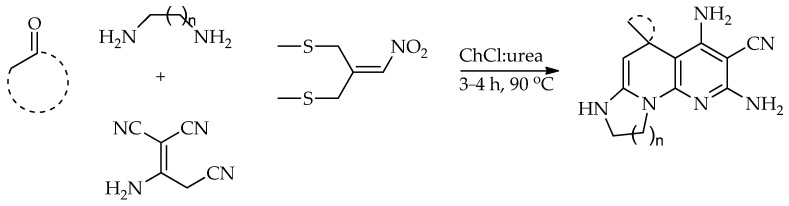
The preparation of polyfunctionalized naphthyridines via a domino four-component reaction in ChCl:urea [63].

**Scheme 9:**
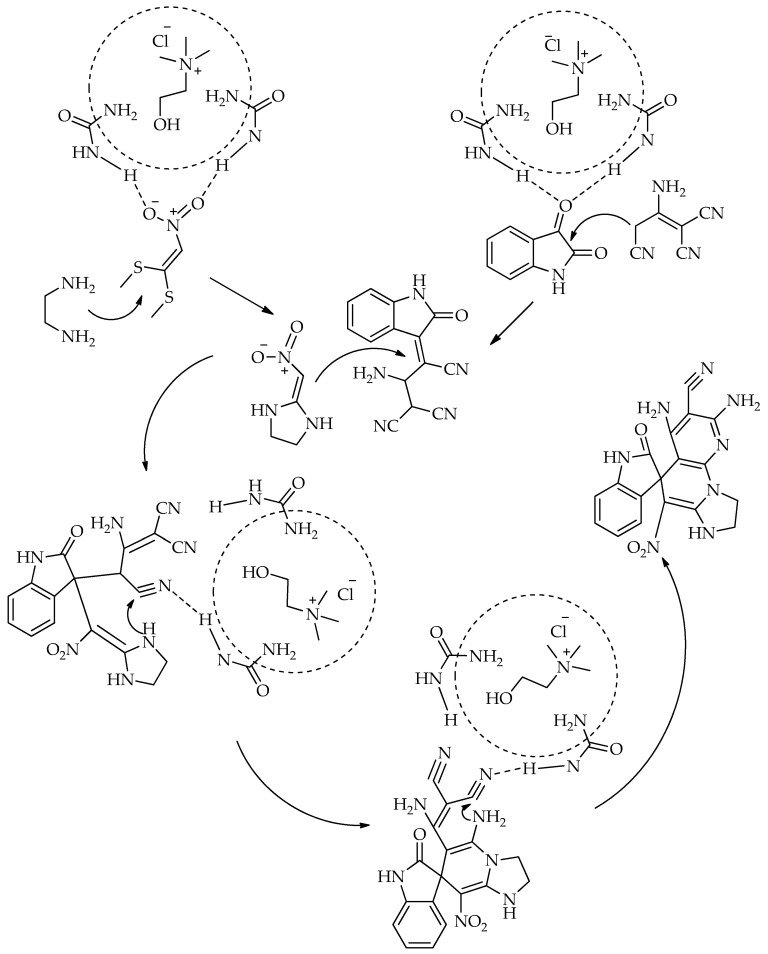
The proposed pathway for the synthesis of polyfunctionalized naphthyridines in ChCl:urea [63].

Shaabani et al. [63] reported a green and sustainable approach for the synthesis of polyfunctionalized naphthyridines via a domino four-component reaction conducted in the DES ChCl:urea. Fully substituted naphthyridines were synthesized from various diamines (including ethylenediamine, propane-1,2-diamine, and 1,2-diaminocyclohexane), 1,1-bis(methylthio)-2-nitroethylene, 2-aminoprop-1-ene-1,1,3-tricarbonitrile, and diverse carbonyl compounds such as benzaldehyde, isatin, ninhydrin, and naphthyl-2-carbaldehyde (Scheme 8). The reaction proceeded efficiently under mild, base-free, and catalyst-free conditions. In this protocol, the DES was proposed to serve a dual role: acting both as a solvent and a catalyst, which activates the carbonyl and cyano groups via hydrogen bonding (Scheme 9). This diversity-oriented approach enabled the efficient construction of the naphthyridine core through a one-pot domino process, leading to a new class of fused, polyfunctionalized naphthyridines. The methodology allowed the simultaneous formation of three new rings and six σ-bonds under mild, base-free conditions.

**Scheme 10:**
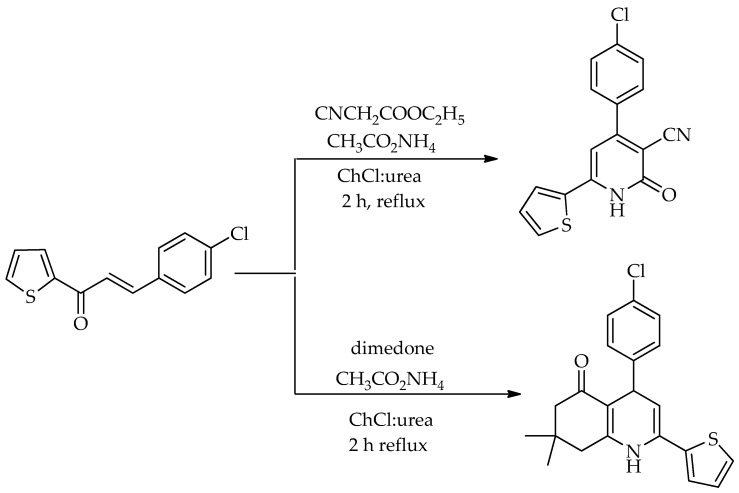
Synthesis of 4-(4-chlorophenyl)-2-oxo-6-(thiophen-2-yl)-1,2-dihydropyridine-3-carbonitrile and 4-(4-chlorophenyl)-7,7-dimethyl-2(thiophen-2-yl)-4,6,7,8-tetrahydroquinolin 5(1*H*)-one in DES [64].

A series of chalcones were synthesized using ChCl-based DES and used as starting materials for the synthesis of various heterocycles, including pyridines [64] (Scheme 10). Chalcone synthesis was carried out in three different DESs. The highest yield (93%) was obtained using ChCl:urea (1:2) in 30 min, while the yield with ChCl:glycerol was lower (87%) even after 2 h of reaction, and with ChCl:oxalic acid it was 79% after 1 h.

**Scheme 11:**
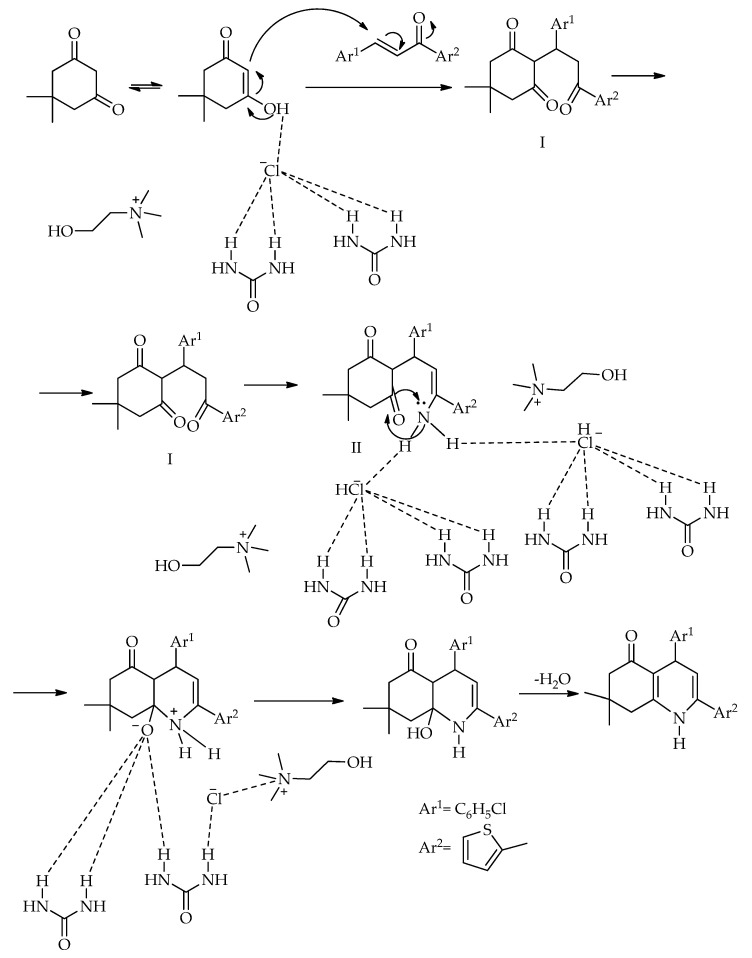
The proposed mechanism for the synthesis of 4-(4-chlorophenyl)-7,7-dimethyl-2-(thiophen-2-yl)-4,6,7,8-tetrahydroquinolin-5(1*H*)-one using a DES [64].

The proposed mechanism for the synthesis of 4-(4-chlorophenyl)-7,7-dimethyl-2-(thiophen-2-yl)-4,6,7,8-tetrahydroquinolin-5(1*H*)-one using a DES involves an initial Michael addition reaction between chalcone and dimedone, yielding intermediate I. This intermediate then condenses with ammonium acetate to form intermediate II. Subsequently, the carbonyl (C=O) group is attacked by the NH_2_ group acting as a nucleophile, followed by the elimination of a water molecule, resulting in the formation of the product (Scheme 11).

**Scheme 12:**
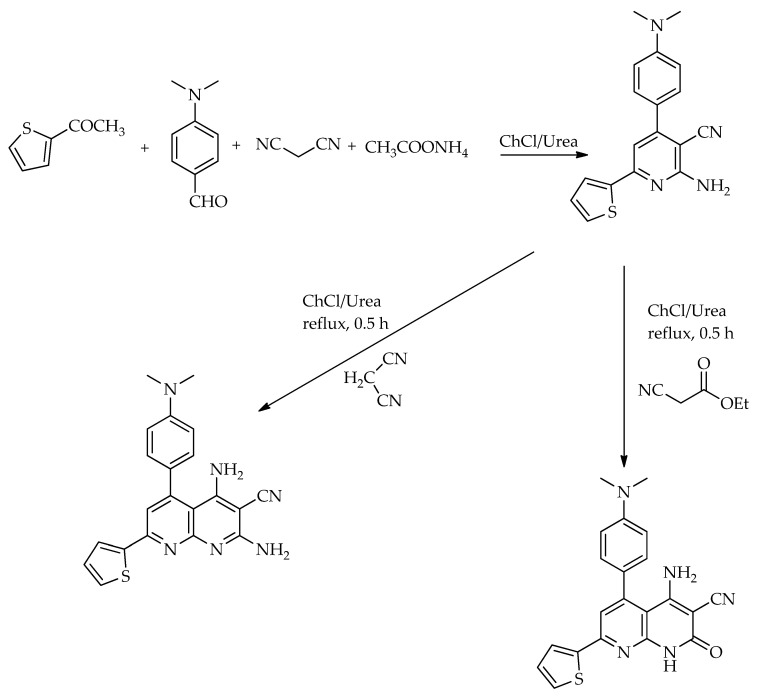
Synthesis of 2-amino-4-(4-(dimethylamino)phenyl)-6-(thiophen-2-yl)nicotinonitrile and derivatives [65].

Mohamed et al. [65] developed a straightforward and environmentally benign strategy for the synthesis of pyrimidines and pyridines using multicomponent reactions in a DES composed of ChCl:urea (1:2). In this DES medium, a Biginelli-type reaction between aromatic aldehydes, 1,3-diphenyl-1,3-propanedione, and thiourea efficiently afforded pyrimidines in high yields (up to 96%) within 30 min. Additionally, a one-pot reaction of 2-acetylthiophene, 4-(dimethylamino)benzaldehyde, and malononitrile in the presence of ammonium acetate, carried out in the same ChCl:urea system, led to the formation of an aminonicotinonitrile derivative 2-amino-4-(4-(dimethylamino)phenyl)-6-(thiophen-2-yl)nicotinonitrile (Scheme 12). The fusion of compound 2 with active methylene compounds such as ethyl cyanoacetate or malononitrile in ChCl:urea under reflux for 25 min resulted in the formation of two compounds, 4-amino-5-(4-(dimethylamino)phenyl)-2-oxo-7-(thiophen-2-yl)-1,2-dihydro-1,8-naphthyridine-3-carbonitrile and 2,4-diamino-5-(4-(dimethylamino)phenyl)-7-(thiophen-2-yl)-1,8-naphthyridine-3-carbonitrile, in high yield (90% and 85%) (Scheme 12).

**Scheme 13:**
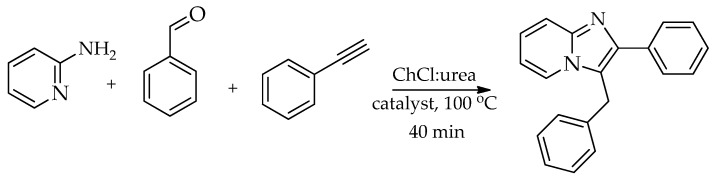
Synthesis of 3-benzyl-2-phenylimidazo [1,2-*a*]pyridine [66].

ChCl:urea DES was used in the synthesis of imidazo [1,2-*a*]pyridines with a Cu(II)-functionalised magnetic Fe_3_O_4_@SiO_2_ catalyst. Optimization of the catalytic performance showed that Fe_3_O_4_ NPs, Fe_3_O_4_@SiO_2_m, and Fe_3_O_4_@SiO_2_-TCCP under reflux in ethanol produced either no product or low yields (0–16%), indicating low catalytic activity. However, the introduction of Fe_3_O_4_@SiO_2_-TCCP-Cu(II) significantly increased reaction efficiency, affording an 86% yield after 90 min. Further optimization using the same catalyst with DES ChCl:urea at 100 °C for 40 min provided the maximum yield (97%), representing the most effective condition. Solvent screening revealed that ethanol gave moderate to high yields, while water, PEG, dioxane, and toluene resulted in very low yields (14–26%) compared to ChCl:urea (Scheme 13) [66].

**Scheme 14:**
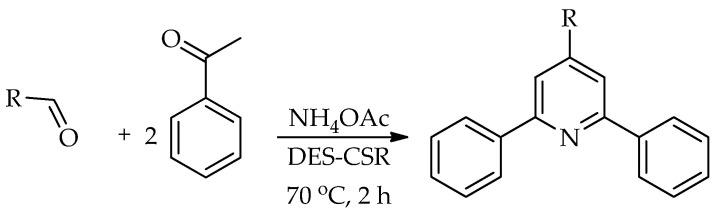
One-step synthesis of 2,4,6-triarylpyridine in DES [67].

In the work of the Kamble and Shankarling [67], an energy-efficient and environmentally friendly pseudo MCR, one-step synthesis of 2,4,6-triarylpyridines is described by combining a DES and concentrated solar radiation (CSR). The different acidic DESs ChCl:oxalic acid, ChCl:malonic acid, ChCl:tartaric acid and ChCl:ZnCl_2_ were tested. The best yield of 78% was achieved in DES ChCl:malonic acid within 4 h. DES ChCl:malonic acid acts as both a solvent and a catalyst, eliminating the need for additional catalysts and volatile organic solvents, while simplifying the synthesis procedure. The integration of DES with CSR significantly reduces the reaction time and saves more than 50% energy compared to conventional heating, which enables good product yields in accordance with the principles of green chemistry (Scheme 14).

**Scheme 15:**
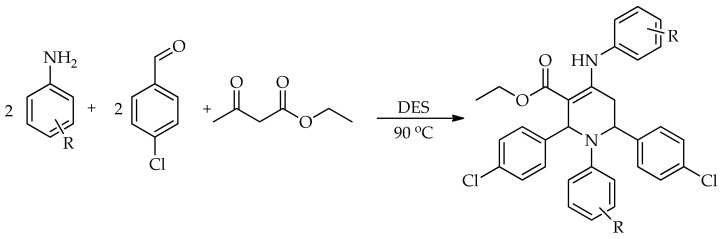
Reaction of *β*-ketoester, aniline, and aldehyde in DES [68].

Goudarzi et al. [68] used DES prepared by a mixing of ethyl triphenylphosphonium bromide (ETPP-Br) and tetrahydrofuran-2,3,4,5-tetra-carboxylic acid as the catalyst for the synthesis of tetrahydropyridines and 1,3-thiazolines-4-ones in pseudo MCR. The compounds were synthesized from the reaction of *β*-ketoester, aniline, and aldehyde at 80 °C in the DES with high yields (60–95%) and appropriate reaction times (1–4 h) (Scheme 15).

**Scheme 16:**
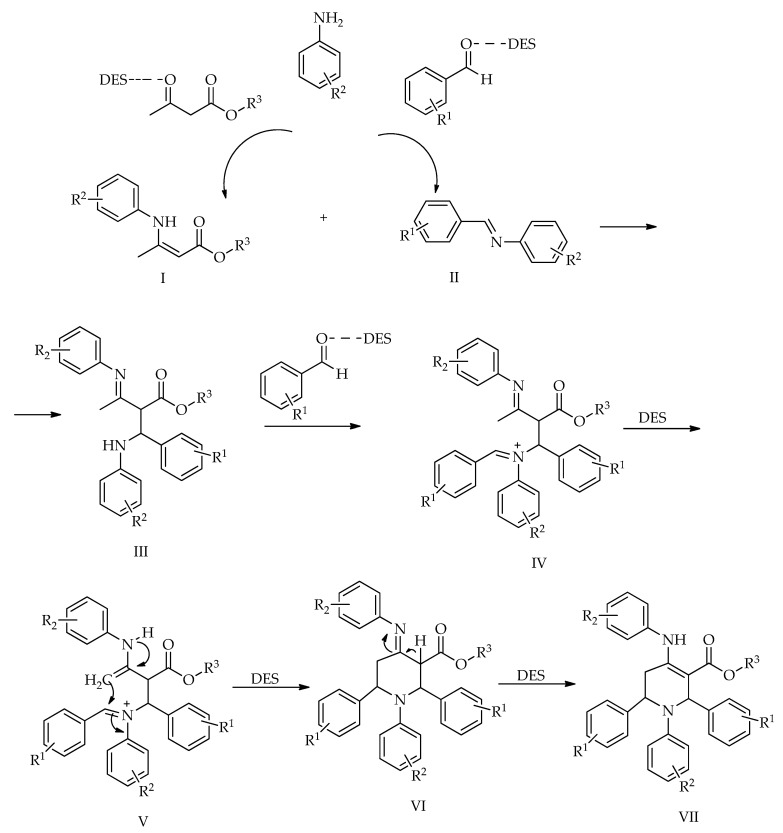
Proposed mechanism for synthesis in DES [68].

The possible mechanism for the synthesis involves the initial reaction of the *β*-keto-ester and aldehyde with aniline in the presence of the DES, producing the enamine (I) and imine (II). Subsequently, the enamine (I) and imine (II) react to form intermediate (III) via an intermolecular Mannich-type reaction. Intermediate (III) then reacts with another molecule of aldehyde to generate intermediate (IV). The tautomerization of (IV) produces intermediate (V), which immediately undergoes an intramolecular Mannich-type reaction to yield intermediate (VI). In the final step, the tautomerization of intermediate (VI) generates the product (Scheme 16).

**Scheme 17:**
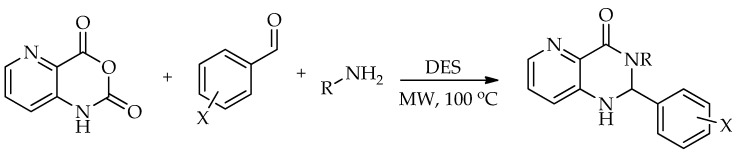
Preparation of monosubstituted pyridopyrimidines in DES [69].

The efficient one-pot multicomponent synthesis of pyridopirimidines was conducted in a DES. In the model reaction, various DESs were tested. In DES benzalkonium chloride:glycerol and benzalkonium chloride:ethylene glycerol, the product was obtained in low yields (52% and 49%). Benzalkonium chloride:urea and benzalkonium chloride:oxalic acid gave better yields (61% and 72%). When the reaction was performed in urea:CuCl_2_, the desired product was obtained with excellent yield (96%) in short reaction time (2 h). A mixture of urea: CuCl_2_ was then used as an efficient catalyst to synthesize different pyridopirimidines using microwave irradiation with excellent yield in a very short time (3–5 min) [69] (Scheme 17). This DES was recycled and reused for four consecutive rounds without showing any significant loss of performance.

**Scheme 18:**
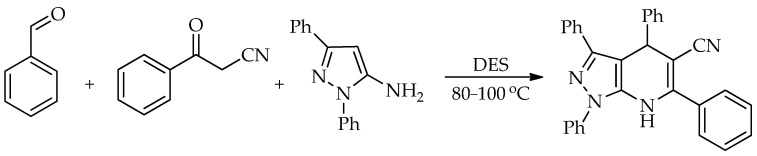
Reaction of benzaldehyde, 3-oxo-3-phenylpropanenitrile and 1,3-diphenyl-1*H*-pyrazol-5-amine [70].

Gao et al. demonstrated that a DES based on L-proline and oxalic acid can act as a highly efficient catalyst for synthesizing 4,7-dihydro-1*H*-pyrazolo [3,4-*b*]pyridine-5-carbonitriles [70]. The reaction is performed as a straightforward one-pot, three-component MCR synthesis using aldehyde, 3-oxopropanenitrile and 1*H*-pyrazol-5-amine (Scheme 18). The method is notable for its broad substrate scope, ease of implementation, catalyst recyclability, and suitability for scaling up to multi-gram quantities. Various aldehydes and 3-oxopropanenitriles can be used successfully, resulting in a significant structural diversity of products with good to excellent yields (72–95%). The catalyst can be reused at least five times without significant loss of catalytic activity.

**Scheme 19:**
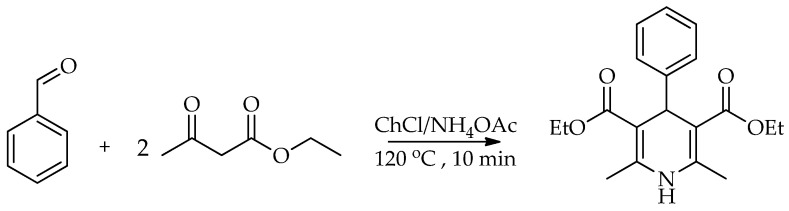
Synthesis of dihydropyridine derivative [71].

The work of Kumar et al. [71] deals with the development of a green and environmentally friendly method for the synthesis of 1,4-dihydropyridine using a DES in the Hantzsch reaction pseudo MCR (Scheme 19). The authors synthesized a new DES system from ChCl and ammonium acetate (NH_4_OAc) in a 1:2 ratio and showed that it has a triple role in the reaction: it acts as a green solvent, as a metal-free catalyst, and as an in situ substrate. Ammonium acetate reacts as an HBD in the eutectic mixture and as a substrate in the reaction. The method enables high product yields (up to 95%), short reaction times (about 10 min), simple product isolation, solvent recycling, and reduced environmental impact compared to conventional methods. The research confirms that the application of the DES system represents a more efficient and environmentally friendly alternative to traditional methods of 1,4-dihydropyridine synthesis, with better efficiency according to green chemistry principles (atom economy, reaction mass efficiency, etc.).

**Scheme 20:**
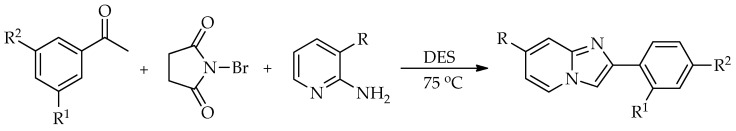
One-pot synthesis of imidazo [1,2-α]pyridines [72].

The research of Shaikh and Kasim [72] describes the development of an environmentally friendly and efficient method for the synthesis of imidazo [1,2-*a*]pyridines using a DES (Scheme 20). The aim of the work was to develop a fast, simple and “green” one-pot method for their synthesis, avoiding toxic solvents and demanding reaction conditions. Therefore, the research methodology involved the condensation of substituted acetophenones, *N*-bromosuccinimide and 2-aminopyridine in a DES prepared from ChCl, urea and thiourea (ratio 1:1:1) whereby α-bromoketone was formed in situ (within one minute at room temperature), after which the addition of 2-aminopyridine and heating to 75 °C gave the target imidazo [1,2-*a*]pyridine. The reaction time was 5–7 min, with high yields of 92–95% obtained under mild reaction conditions. Therefore, the authors state that the advantages of this green synthesis are short reaction time, high yields, simple product processing, and avoidance of hazardous solvents and catalysts, which is in line with the principles of green chemistry.

**Scheme 21:**
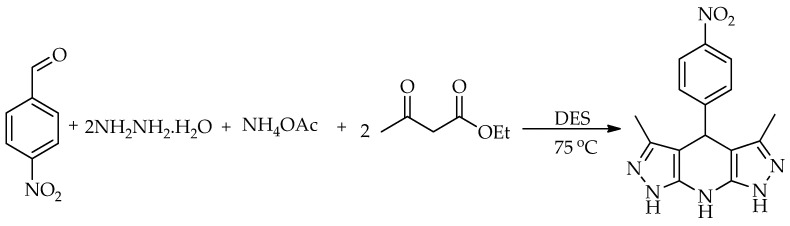
One-pot synthesis of tetrahydrodipyrazolopyridines [73].

Mohammad et al. [73] designed the efficient one-pot synthesis of chromeno [4,3-*b*] chromenes and tetrahydrodipyrazolopyridines using ETPP-Br/THF-TCA-DES (ethyl triphenylphosphonium bromide and tetrahydrofuran-2,3,4,5-tetracarboxylic acid) as a green and reproducible catalyst (Scheme 21). A diverse set of compounds were produced with high yields (86–98%) in short times (9–18 min).

**Scheme 22:**
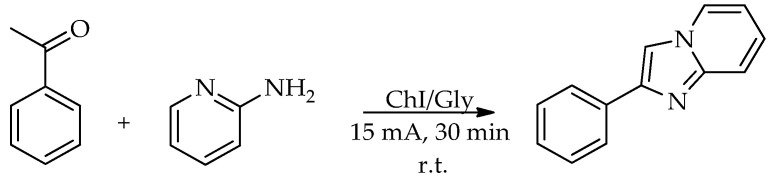
Synthesis of 2-phenylimidazo [1,2-*a*]pyridine in ChI:Gly DES [74].

Karamifar et al. [74] investigate the mechanism of electro-iodination using a ChI:Gly DES, which functions multifunctionally as solvent, electrolyte, and catalyst in combination with inexpensive iron and graphite electrodes, enabling a green and cost-effective synthetic approach. In the first step, an efficient synthesis of 2-iodo-1-phenylethanones was achieved in high yields (91–97%), followed by the application of the same electrochemical system to the one-pot synthesis of 2-phenylimidazo [1,2-*a*]pyridines (Scheme 22). The work emphasizes the sustainable use and reusability of DESs, mild reaction conditions, reduced waste and energy consumption, and mechanistic validation (radical pathway and in situ I_2_ generation), contributing to the advancement of green electro-organic synthesis.

**Figure 3:**
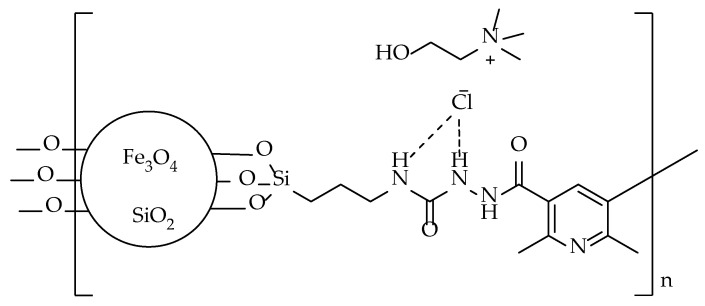
DES supported on Fe_3_O_4_@SiO_2_ (catalyst II) [75].

A DES supported on Fe_3_O_4_@SiO_2_ (catalyst II) (Figure 3) was applied as a catalyst for the one-pot synthesis of pyrazolopyridines under mild reaction conditions [75]. The multicomponent reaction of aryl aldehydes, ammonium acetate, hydrazine monohydrate, and ethyl acetoacetate with 10 mg of catalyst II under solvent-free conditions at 25 °C produced the desired product in high yield (81–92%). The recovery and reuse of catalyst II were studied and evaluated using a model reaction. The synthesis can be repeated five times with only a negligible decrease in yield.

**Scheme 23:**
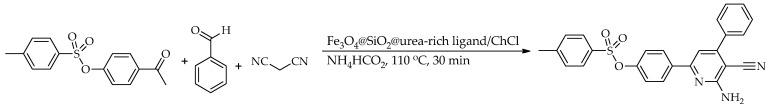
Synthesis of 4-(6-amino-5-cyano-4-phenylpyridin-2-yl)phenyl 4-methylbenzenesulfonate by using Fe_3_O_4_@SiO_2_@urea-riched ligand/ChCl [76].

A new heterogeneous catalytic system was developed by modifying urea-functionalised magnetic nanoparticles with choline chloride [Fe_3_O_4_@SiO_2_@urea-rich ligand/ChCl], and used for the synthesis of hybrid pyridines containing sulfonate and/or indole moieties [76] (Scheme 23).

The catalyst was successfully applied in an MCR under optimal solvent-free conditions at 110 °C, achieving high product yields of 75% to 90% within short reaction times of 20 to 50 min. A key advantage of this system is its excellent reusability, as the catalyst can be easily separated using an external magnet and reused up to five times without significant loss of activity.

## 3. Synthesis of Coumarins Using DESs

**Scheme 24:**
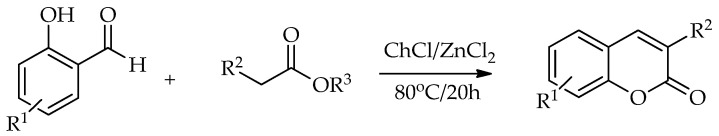
Reaction of substituted salicylaldehyde and active methylene compounds [78].

**Scheme 25:**
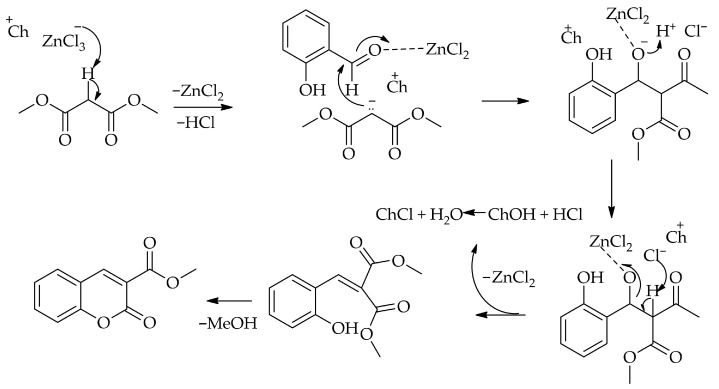
Proposed mechanism in reaction of substituted salicylaldehyde and active methylene compounds [78].

A simple Knoevenagel condensation to synthesize coumarins can be performed from salicylaldehyde and carbonyl compounds containing an active methylene group. A few studies were published dealing with this condensation in DESs. Lončarić et al. [77] performed a coumarin synthesis from substituted salicylaldehydes and different active methylene compounds (ethyl acetoacetate, ethyl cyanoacetate, dimethyl malonate, diethyl malonate, and ethyl benzoylacetate) using different DESs. A screening experiment on 20 different DESs revealed that ChCl:urea was the most effective DES for this synthesis, using a temperature of 80 °C. Keshavarzipour and Tavakol [78] performed the same reaction using a ChCl:ZnCl_2_ DES in the reaction of substituted salicylaldehyde and active methylene compounds (dimethyl malonate, ethyl cyanoacetate and ethyl 3-oxo-3-phenylpropanoate) (Scheme 24). A DES composed of ChCl:SnCl_2_ gave lower product yields. They speculate that the acidic nature of the DES is the cause of the catalytic activity, where zinc chloride is Lewis acid, while Cl^−^ takes a proton of diethylmalonate (Scheme 25).

**Scheme 26:**
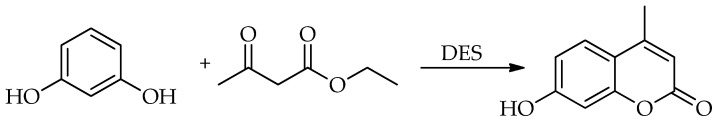
Reaction of resorcinol and ethyl acetoacetate in DES [79].

Mohammad et al. [79] reacted resorcinol and ethyl acetoacetate using acidic DES composed of *p*-toluenesulfonic acid and benzyl dimethyl (2-hydroxyethyl) ammonium chloride, to obtain coumarins under optimized conditions in 120 min at 100 °C (Scheme 26).

**Scheme 27:**
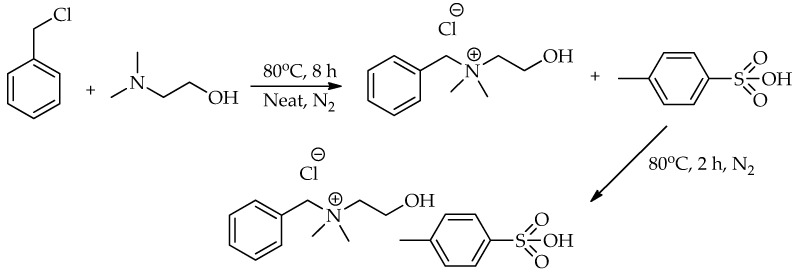
Preparation of acidic DESs [79].

They synthesized different acidic DESs, but the one formed by using benzyl chloride, 2-(dimethylamino)ethanol and *p*-TSA was the most effective (Scheme 27).

**Scheme 28:**
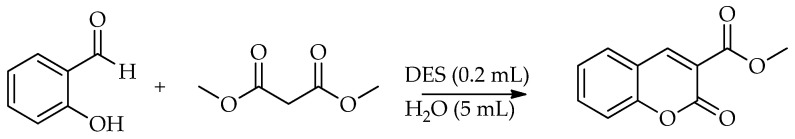
Reaction of salicylaldehyde and *β*-ketoesters in DES [79].

The same authors performed a synthesis of 3-substituted-coumarins in reaction of salicylaldehyde and *β*-ketoesters. This reaction proceeded efficiently in water with the addition of the DES at room temperature, where piperidine:acetic acid DES was the most effective and the products were obtained in 5–15 min (Scheme 28) [79].

**Scheme 29:**
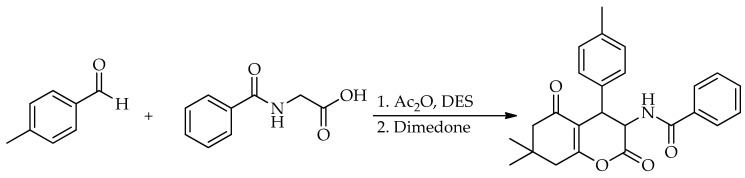
Synthesis of 3-aminohexahydrocoumarins [80].

**Scheme 30:**
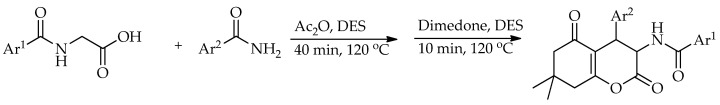
Optimal conditions for synthesis of 3-aminohexahydrocoumarins [80].

Interesting research on a combination of microflow technology and DESs for the synthesis of 3-aminohexahydrocoumarins was performed by Zamani and Khosropour [80]. Before they applied microflow technology, they examined which DES and conditions are the most effective for the synthesis of the desired compounds. As a model reaction (Scheme 29), they used different choline chloride-based DESs (with benzoic acid, benzamide, glycerol, thiourea and urea) varying the reaction temperature. The most efficient DES was found to be the one composed of ChCl:urea and a temperature of 120 °C. After that, they investigated the multi-step reaction to obtain the same compound in a flow system. Here, the optimal conditions were *T*_1_ = *T*_2_ = 120 °C, with times *t*_1_ = 40 min and *t*_2_ = 10 (Scheme 30). The work-up of this multi-step reaction was also easy and performed in an environmentally acceptable way, while the authors have also proven that the DES could be successfully recycled for few cycles.

**Scheme 31:**
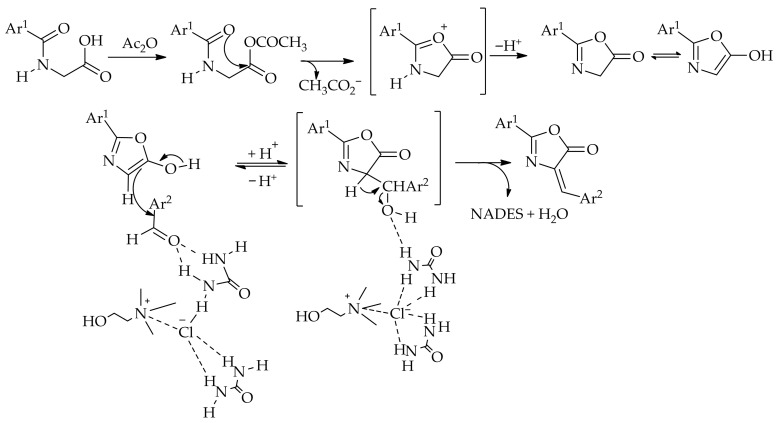
Plausible mechanism step 1 in DES [80].

**Scheme 32:**
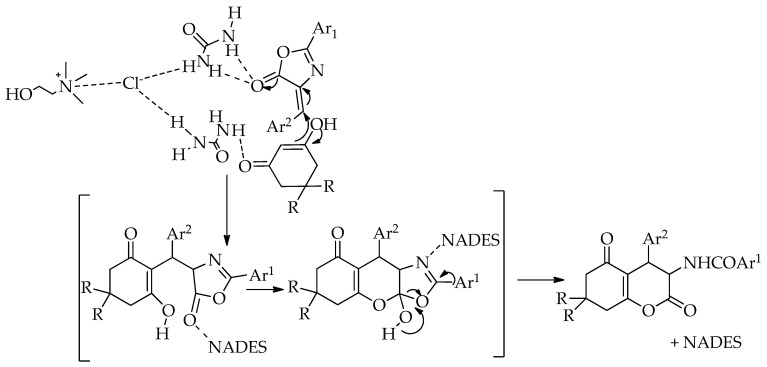
Plausible mechanism step 2 in DES [80].

The authors speculated the plausible mechanism for both step 1 and step 2 (Scheme 31 and Scheme 32) depicts the formation of azlactone from arylaldehyde, acetic anhydride and hippuric acid. The formation of this azlactone is due to the strong hydrogen bonding caused by the DES. Furthermore, in step 2 a formation of the desired product is obtained through a Michael addition/transesterification reaction, where the activation of carbonyl groups in both reactants is also obtained through strong hydrogen bonding due to the presence of the DES.

**Scheme 33:**
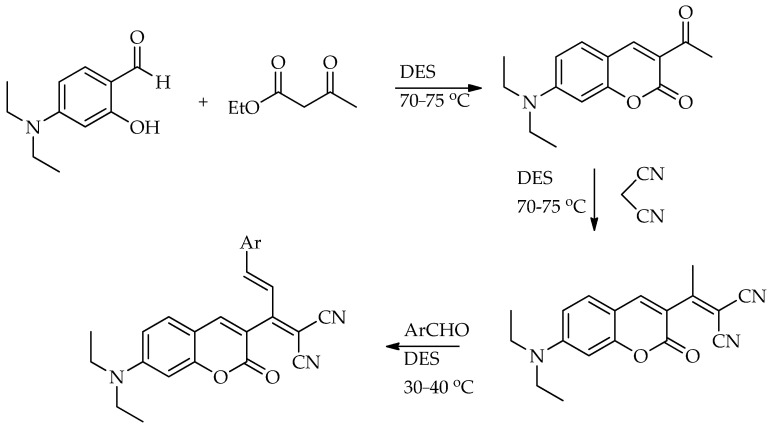
Synthesis of 2-(1-(7-(diethylamino)-2-oxo-2*H*-chromen-3-yl)ethylidene)malononitriles [81].

**Scheme 34:**
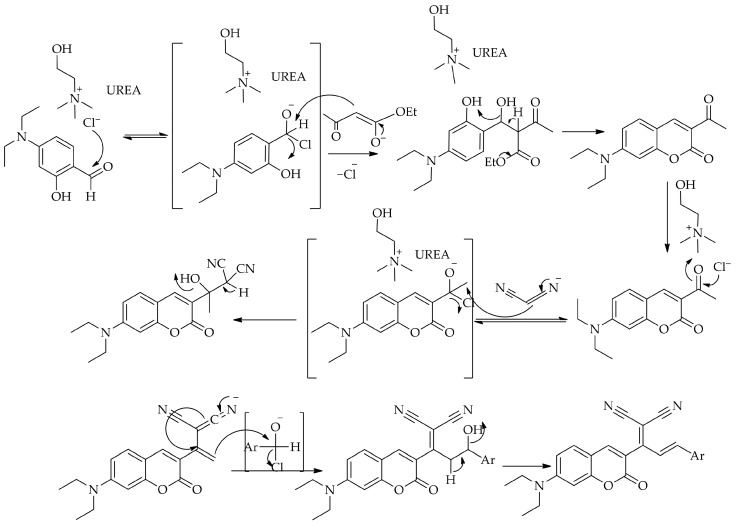
Proposed mechanism for synthesis of 2-(1-(7-(diethylamino)-2-oxo-2*H*-chromen-3-yl)ethylidene)malononitriles [81].

Phadtare et al. [81] developed a green method for the synthesis of 2-(1-(7-(diethylamino)-2-oxo-2*H*-chromen-3-yl)ethylidene)malononitriles (Scheme 33). They used a ChCl:urea DES to perform a one-pot three-step reaction to obtain the desired coumarins. The first two steps were performed over 1–2 h each, using a temperature of 70–75 °C, while the third step was performed at 30–40 °C over 1–2 h. Isolation was simple, where the addition of water precipitated the products and the DES was recovered by phase separation and the subsequent evaporation of the water. They postulate that the acidic nature of the DES corresponds to its catalytic activity (Scheme 34). A reaction where piperidine was used as a catalyst took 12 h and gave lower yields.

Although, to date, no literature has been identified reporting the electrochemical synthesis of coumarins using deep eutectic solvents (DESs), the electrochemical characterization of synthesized coumarins has been conducted. Janeiro et al. (2013) designed, synthesized, and evaluated the electrochemical redox mechanisms of various substituted 3-arylcoumarins to better understand their structure and electrochemical behaviour [82]. The authors investigated the influence of bromine, methyl, or other hydroxyl groups in different positions of the coumarin scaffold, using cyclic, differential pulse, and square wave voltammetry at a glassy carbon electrode at different pH values, and compared their results with differently substituted 8-hydroxy-3-arylcoumarins. Further, Lin et al. [83] performed continuous electrosynthesis of iso-coumarins from *o*-(1-alkynyl) benzoates under metal- and oxidant-free conditions, demonstrating potential for pilot-scale experiments. Although the electrochemical synthesis of coumarins is well established, the solvents used remain conventional, including acetonitrile (MeCN), dimethylformamide (DMF), alcohols, and aqueous buffers. Therefore, the electrochemical synthesis of coumarins in DESs may represent a novelty in the development of electrochemical methods and the synthesis of organic compounds.

### Synthesis of Different Coumarin Core Containing Hybrids

Although in the previous paragraph the synthesis of coumarins was described, some interesting and worth-mentioning research was found on the synthesis of coumarin hybrids with different entities.

**Scheme 35:**
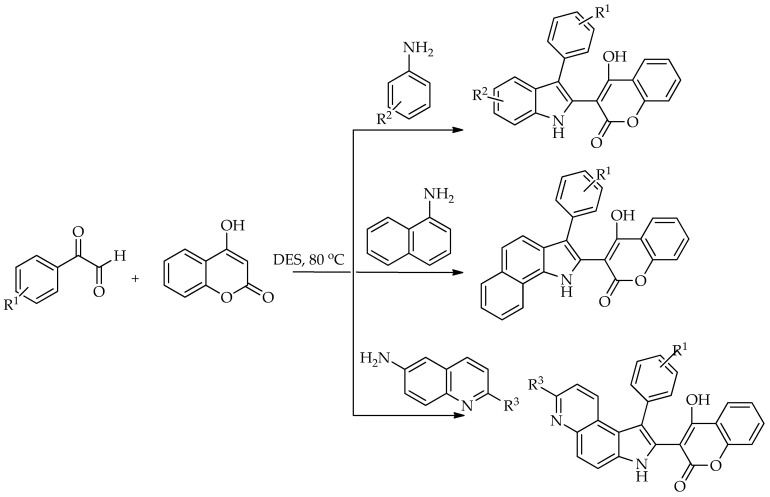
Synthesis of different indole–coumarin hybrids using different DESs [84].

Devi et al. [84] investigated the synthesis of different indole–coumarin hybrids using different DESs. A one-pot model reaction between 4-chlorophenylglyoxal, 4-hydroxycoumarin, and 4-methylaniline was performed in different DESs, where choline chloride was combined with citric acid, zinc chloride, urea, glycerol, ascorbic, malonic, oxalic acid and PTSA (*p*-toluenesulfonic acid). The most effective DES for this synthesis was found to be the one with citric acid, where the reaction time was 4 h and reaction *T* 80 °C. In general, acidic DESs were more efficient than others. The authors speculate that citric acid acidic protons have a predominant influence on both reaction selectivity and reactivity. Not only was this DES applied for the synthesis of 4-hydroxy-3-(3-aryl-1*H*-indol-2-yl)-2*H*-chromen-2-ones, but it was also successfully utilized for the synthesis of 4-hydroxy-3-(3-aryl-1*H*-benzo[g]indol-2-yl)-2*H*-chromen-2-ones as well as 4-hydroxy-3-(1-phenyl-3*H*-pyrrolo [3,2-*f*]quinolin-2-yl)-2*H*-chromen-2-ones (Scheme 35). This method is much less time consuming, since Mehrabi et al. [85] obtained similar compounds using reflux conditions in ethanol with a reaction time of 10 h.

**Scheme 36:**
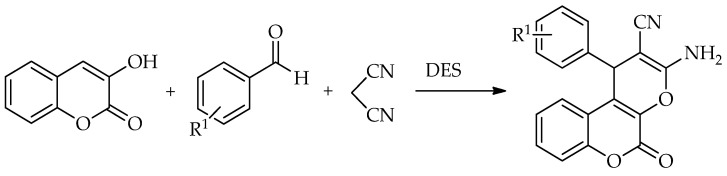
One-pot synthesis of pyrano [3,2-*c*]chromenes from 3-hydroxycoumarin, benzaldehydes and malononitrile [86].

Tasqeeruddin et al. [86] investigated the influence of solvent and solvent-free conditions with the addition of a ChCl:urea:thiourea DES as the catalyst in a one-pot synthesis of pyrano [3,2-*c*]chromenes from 3-hydroxycoumarin, benzaldehydes and malononitrile. Their attempt to apply solvent-free and catalyst-free conditions yielded only trace amounts of the desired products. Furthermore, they added the DES as a catalyst in different solvents (acetonitrile, dichloromethane, ethanol, and PEG-400) which resulted in an increase in the product yield but still was not efficient enough. Then, when water was used, the product yield increased to 80%. Yet, the best yield was obtained in the solvent-free reaction using 20 mol% of catalyst in only 15 min (Scheme 36).

**Scheme 37:**
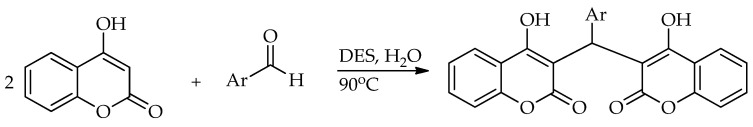
Synthesis of biscoumarins [87].

A DES composed of taurine and ChCl (1:2) was successfully applied as a catalyst in the synthesis of biscoumarins (Scheme 37). Their attempt to perform a solvent-free reaction, without and with a catalyst, produced only a trace amount of the desired product. When water was used as a solvent, 18 mol% and 20 mol% of the catalyst yielded almost quantitative amounts of the product, while the reaction time was 20–22 min and with temperatures of 90 °C or reflux conditions. Using solvents such as ethanol, acetonitrile, or tetrahydrofuran gave low yields or trace amounts of the product [87]. Similar compounds were obtained by Lončar et al. [88] using ethanol as a solvent and molecular iodine as a catalyst. An ultrasound-assisted reaction produced desired products in 30 min, while a microwave-assisted reaction was longer and took 90 min. In the research of Tzani et al. [89] biscoumarins were synthesized using ionic liquids at 40 °C, but the reaction was more time consuming and required 3 h to complete. Caffeine can also be used as a catalyst for this reaction. Ghahnavie et al. [90] performed this synthesis in water, using 10 mol% of caffeine at 80 °C for 30 min to obtain biscoumarin in high yields [90].

**Scheme 38:**
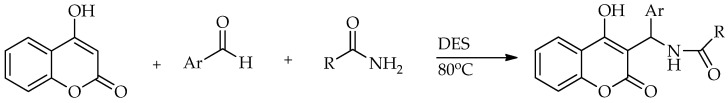
Synthesis of 3-amido-alkyl-4-hydroxycoumarins [91].

ChCl:urea DES was successfully applied for the synthesis of 3-amido-alkyl-4-hydroxycoumarins, in a three-component reaction between 4-hydroxycoumarin, aromatic aldehydes and different amides (Scheme 38). Different DESs were screened for this application; all choline chloride-based in combination with ZnCl_2_, PTSA, citric and oxalic acid and urea, as well as different temperatures 80–100 °C. ChCl:urea (1:2) DES was found to be the most effective, while the reaction was performed at 80 °C for 90–105 min. The authors also compared their work with other authors, showing that this methodology is more efficient [91]. For example, in the research of Anaraki-Ardakanik and Charooseai (2015) ZnO nanoparticles were used as catalyst for the same solvent-free reaction and after 130 min 85% yield was accomplished [92].

**Scheme 39:**
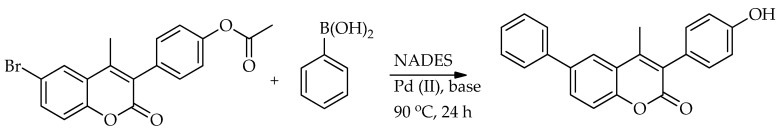
Suzuki–Miyaura cross coupling for the synthesis of coumarins [93].

Interesting research was conducted by Katopodi et al. [93], with even more interesting conclusions. They wanted to optimize the Suzuki–Miyaura cross coupling for the synthesis of substituted coumarins. First, they performed a model reaction in glycerol and different DESs (ChCl:glycerol, betaine:glycerol, L-proline:glycerol, fructose:urea:H_2_O, and glucose:urea:H_2_O), using sodium or potassium carbonate as a base and Pd(OAc)_2_ or PdCl_2_ as a catalyst (Scheme 39). DESs which did not contain glycerol did not yield a desired product, while ChCl:glycerol (1:2) with K_2_CO_3_ and Pd(OAc)_2_ gave high product yield.

**Scheme 40:**
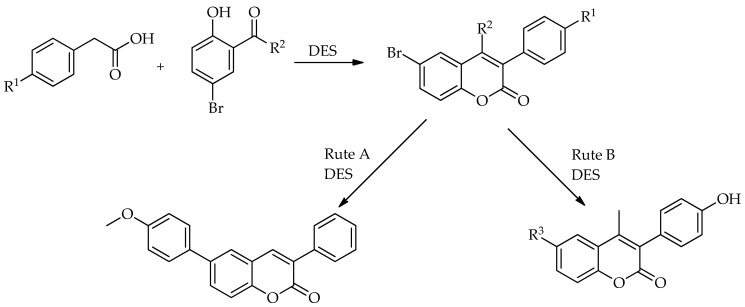
NADES-mediated Suzuki–Miyaura coupling [93].

Their intention was C-C formation, but they noticed that in all glycerol-based DESs and pure glycerol, deacetylation occurred. They investigated this reaction further, using ChCl:glycerol or pure glycerol, and K_2_CO_3_. They applied conventional heating and ultrasound irradiation, showing that the reaction time could be decreased if ultrasound is applied. For the whole synthetic procedure, they also examined two reaction routes. One included the addition of all the reactants at once, and the other was a two-step reaction: first deacetylation was performed and then catalyst and boronic acid were added (Scheme 40). The second protocol gave better product yields. Furthermore, they also noticed the formation of palladium nanoparticles in ChCl:glycerol (1:2) and ChCl:betaine (1:2) DES [93].

## 4. Synthesis of Quinazolinones in DESs

**Scheme 41:**
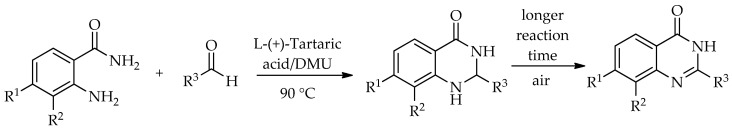
DES-mediated cyclization of anthranilamides with aldehydes [94].

An important early contribution in this area was reported by Ghosh and Nagarajan [94], who showed that the condensation of anthranilamides with aldehydes in an L-(+)-tartaric acid/1,3-dimethylurea (DMU) DES can initially afford 2,3-dihydroquinazolin-4(1*H*)-ones and, under open-air conditions, undergo further oxidation to the corresponding quinazolin-4(3*H*)-ones. In their optimization study, several DES mixtures were examined using anthranilamide and *o*-tolualdehyde as model substrates, and L-(+)-tartaric acid/DMU (3:7) was identified as the most effective medium. Under the optimized conditions, the reaction was performed at 90 °C, and the product distribution was found to depend strongly on both reaction time and the nature of the aldehyde. The method tolerated aromatic, aliphatic, and heteroaromatic aldehydes, furnishing representative quinazolin-4(3*H*)-one and 2,3-dihydroquinazolin-4(1*H*)-one products in good to excellent yields. In the model scope, reaction times ranged from 2 to 24 h, with isolated yields from 25% for mixed quinazolin-4(3*H*)-one/2,3-dihydroquinazolin-4(1*H*)-one outcomes to 92% for 2,3-dihydroquinazolin-4(1*H*)-one formation (Scheme 41). In general, aliphatic aldehydes and aromatic aldehydes bearing electron-donating substituents were more readily converted into quinazolin-4(3*H*)-ones, whereas electron-withdrawing substituents more often remained at the 2,3-dihydroquinazolin-4(1*H*)-one stage even after prolonged reaction times. This study is particularly useful as an opening example because it clearly illustrates the close relationship between the two scaffold families and shows that DES-mediated quinazolinone synthesis can be governed not only by substrate scope but also by control of the oxidation state.

**Scheme 42:**
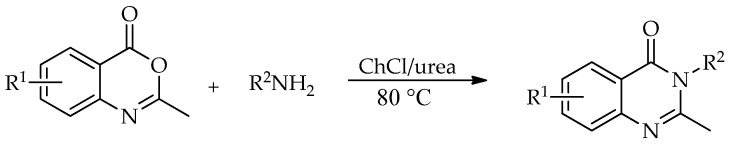
Synthesis of substituted quinazolin-4(3*H*)-ones [95].

**Scheme 43:**
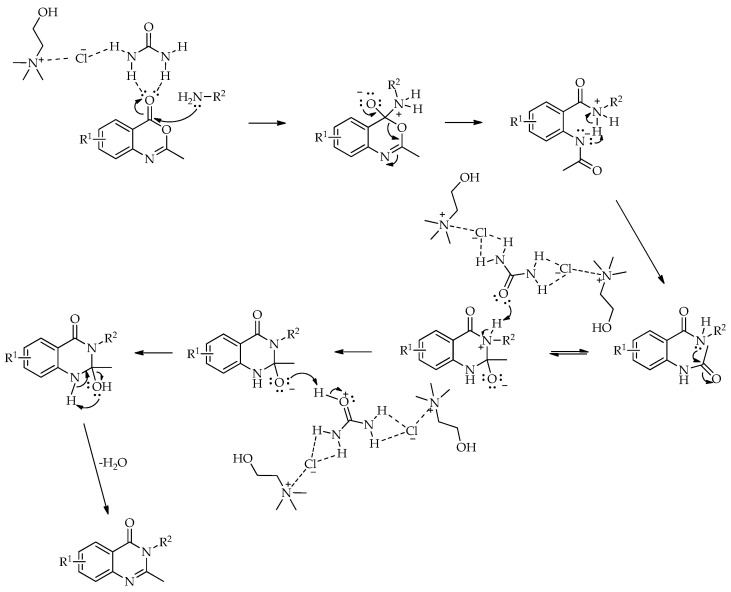
A possible mechanism for the synthesis of substituted quinazolin-4(3*H*)-ones promoted by ChCl:urea [96].

A different route to quinazolin-4(3*H*)-ones was reported by Komar et al. via a benzoxazinone intermediate [95]. In this study, 2-methyl-3-substituted quinazolin-4(3*H*)-ones were obtained by the cyclization of preformed benzoxazinone intermediates with the corresponding amines in ChCl:urea (1:2) at 80 °C (Scheme 42). Although no extensive DES screening was conducted, the chosen medium enabled the synthesis to proceed under relatively mild conditions, without an additional catalyst, and allowed simple product isolation by water addition and filtration. Using this protocol, six derivatives were prepared in 53–84% isolated yields. For some substrates, molecular sieves were required to suppress benzoxazinone hydrolysis and facilitate cyclization, while the authors proposed that the hydrogen-bonding network of the DES stabilizes the ring-opened intermediate and promotes the closure of the quinazolinone ring (Scheme 43).

The same strategy was later developed by the same group [96]. In that study, the earlier approach was expanded into a broader and more systematic investigation, in which twenty ChCl-based NADESs were screened using 2-methylbenzoxazin-4-one and 4-chloroaniline as model substrates. Among the tested systems, ChCl:urea again proved to be the most suitable medium, giving the model product in 61% yield after 60 min at 80 °C. Subsequent optimization showed that both temperature and activation mode had a pronounced effect on the reaction outcome. Under conventional heating, the best yield for the model reaction increased to 68% after 240 min, whereas microwave irradiation remained less effective even after longer reaction times. Ultrasound provided a moderate improvement, while mechanochemical conditions proved to be the most efficient, affording the model product in 71% yield after only 20 min using a reduced amount of ChCl:urea. The optimized procedure was then applied to a broader set of amines and hydrazides, furnishing 29 quinazolin-4(3*H*)-ones overall. Across the substrate scope, conventional heating gave the target compounds in 17–73% yields, ultrasound in 15–56%, microwave irradiation in 10–41%, and mechanochemistry in 45–87% yields, confirming mechanochemistry as the most effective of the examined green techniques; see Scheme 43.

The transformation of 2-methylbenzoxazin-4-one into quinazolin-4(3*H*)-ones can be rationalized by the sequence of steps shown in Scheme 43. A nucleophilic attack of the amine on the carbonyl group of 2-methylbenzoxazin-4-one leads to ring opening and the formation of a 2-acetamido-*N*-substituted benzamide intermediate. The observation of two by-products in the initial study supports the reversible nature of this step, associated with intramolecular cyclization processes. Subsequently, ChCl/urea facilitates proton transfer steps, including the deprotonation of the positively charged nitrogen and the protonation of the oxygen at C-2, thereby activating the system for further transformation. In the final step, the elimination of water occurs, accompanied by the formation of a C=N double bond, leading to the quinazolin-4(3*H*)-one.

**Scheme 44:**
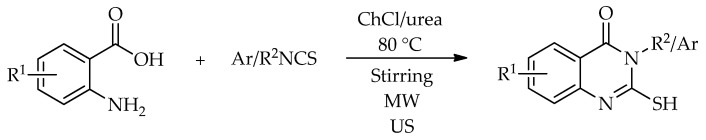
Synthesis of substituted 2-mercaptoquinazolin-4(3*H*)-ones by green methods [96].

**Scheme 45:**
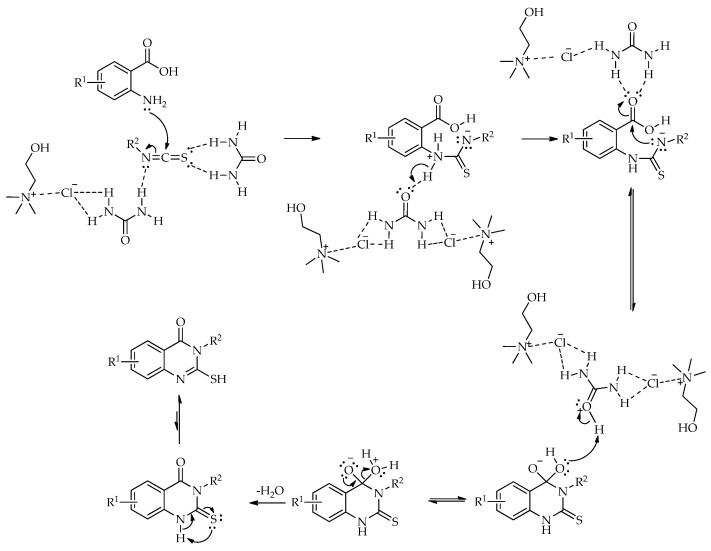
A plausible mechanism for the synthesis of substituted 2-mercaptoquinazolin-4(3*H*)-ones [96].

Within the same broader quinazolinone family, Komar et al. also reported the synthesis of 2-mercaptoquinazolin-4(3*H*)-ones from five anthranilic acids and aliphatic or aromatic isothiocyanates [96]. In their study, the model reaction of anthranilic acid with phenyl isothiocyanate was first examined in ChCl:urea (1:2) at various temperatures and reaction times, with the best result (63%) obtained at 80 °C after 2 h. Subsequent screening of twenty ChCl-based DESs again identified ChCl:urea (1:2) as the most suitable medium, whereas several acidic DESs did not yield the target product under the conditions tested. Using the optimized protocol, the authors prepared a series of 55 substituted 2-mercaptoquinazolin-4(3*H*)-ones in generally moderate to good yields (Scheme 44). The same solvent system was then evaluated under stirring, microwave, and ultrasound conditions. Stirring at 80 °C proved the most effective, while microwave irradiation was the least efficient. This study is particularly important as it demonstrates that acidic DESs are not necessarily preferable in quinazolinone synthesis (Scheme 45).

A plausible reaction mechanism for the formation of substituted quinazolin-4(3*H*)-ones is presented in Scheme 45. The reaction is initiated by a nucleophilic attack of the amino group of anthranilic acid on the electrophilic carbon of the isothiocyanate, leading to the formation of a thiourea intermediate. Subsequently, intramolecular cyclization occurs via a nucleophilic attack of the nitrogen onto the thiocarbonyl carbon, resulting in ring closure and the formation of the quinazolinone framework. This step is followed by proton transfer and rearrangement processes leading to the final product. The ChCl:urea DES facilitates the reaction through hydrogen bond interactions, enhancing the electrophilicity of the isothiocyanate and stabilizing key intermediates.

**Scheme 46:**
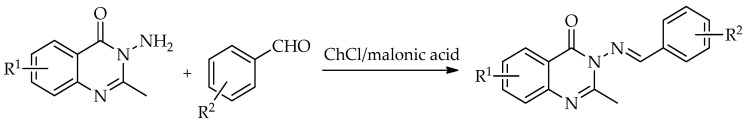
Synthesis of quinazolinone Schiff bases [98].

A related strategy was explored for the synthesis of quinazolinone Schiff bases from 3-amino-2-methylquinazolin-4(3*H*)-ones and aromatic aldehydes. Screening twenty ChCl-based DESs revealed a clear preference for systems containing acidic components, with ChCl:malonic acid (1:1) giving the best result for the model reaction (96% yield) [97]. This trend is consistent with the conventional preparation of Schiff bases, where acid catalysis facilitates carbonyl activation and the dehydration of the intermediate carbinolamine [98]. Further optimization identified 80 °C, 30 min, and 10 mL of DES as the optimal conditions for the conventional procedure, while a comparison of green activation modes again showed mechanochemistry to be the most efficient approach. Using the optimized conditions, a total of 40 quinazolinone Schiff bases were prepared, with isolated yields of 63–97% under conventional heating, 39–68% under microwave irradiation, 40–80% under ultrasound, and 79–97% under mechanochemical conditions. The selected DES also showed good reusability in the model reaction, with the isolated yield decreasing only gradually from 97% in the initial run to 95%, 94%, and 91% over the first three cycles, followed by a more pronounced drop to 83% in the fourth cycle (Scheme 46).

**Scheme 47:**
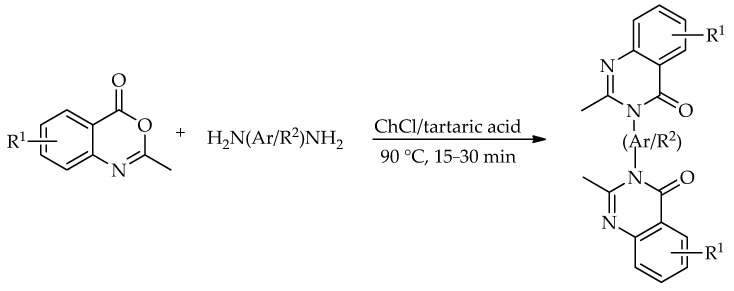
Synthesis of bis-quinazolinones in DES [99].

Ultrasound-assisted synthesis was also successfully applied to the preparation of bis-quinazolin-4(3*H*)-ones from 2-methylbenzoxazin-4-ones and diamines, using ChCl:L-(+)-tartaric acid as the reaction medium. Optimization studies demonstrated a clear advantage of the DES over conventional solvents and other tested systems, with the model reaction achieving a 99% yield after 15 min of sonication at 90 °C. The method was subsequently applied to a wide range of substituted benzoxazinones and both aliphatic and aromatic diamines, affording the target bis-quinazolinones in generally high to excellent yields, with halogen-substituted substrates particularly well tolerated (Scheme 47). The protocol was also demonstrated on a gram scale, and the DES could be reused five times, with only a gradual decrease in isolated yield from 99% to 96%. This study is especially valuable in the context of a review, as it goes beyond the substrate scope to include recyclability, green metrics, and biological evaluation of the synthesized products [99].

**Scheme 48:**
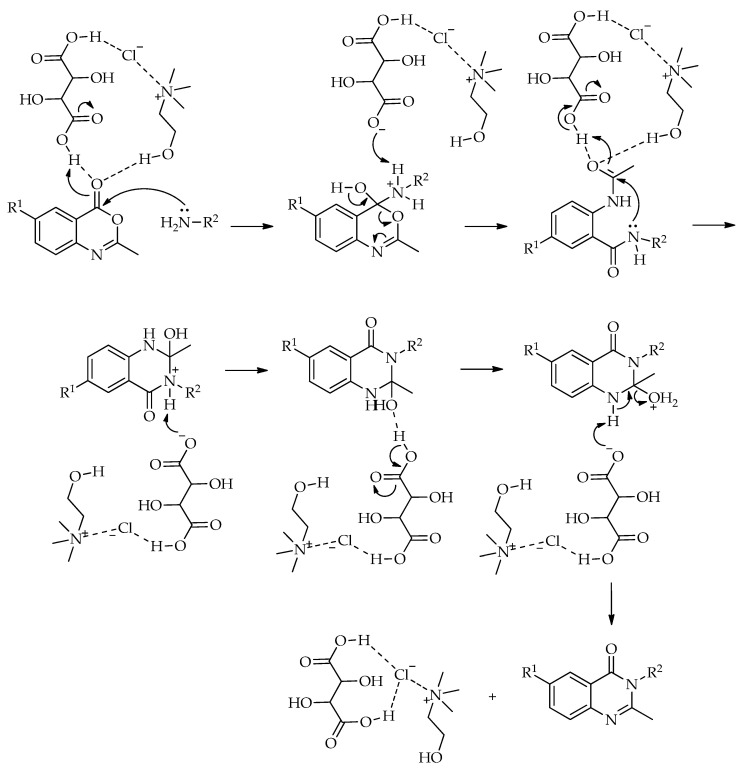
A plausible mechanism for the preparation of bis-quinazolin-4(3*H*)-ones [99].

The formation of bis-quinazolin-4(3*H*)-ones from diamines and benzoxazines can be rationalized by the pathway depicted in Scheme 48. A nucleophilic attack of the amine group (-NH_2_) on the carbonyl carbon of the benzoxazine leads to ring opening and the formation of an amide-type intermediate. This intermediate then undergoes intramolecular cyclization via the nucleophilic attack of the amide nitrogen onto the carbonyl carbon, resulting in the closure of the quinazolinone ring. Subsequent dehydration affords the final bis-quinazolin-4(3*H*)-one product. The L-(+)-tartaric acid: ChCl system plays a dual role, acting both as a reaction medium and as a catalytic system by facilitating proton transfer processes.

**Scheme 49:**
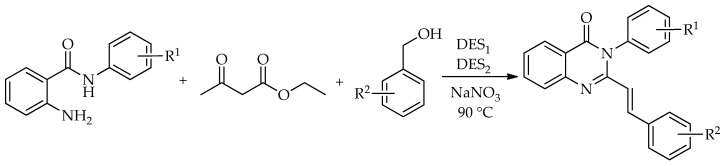
Synthesis of styrylquinazolinon-4(3*H*)-ones [100].

A more recent contribution described the tandem one-pot synthesis of piriqualone and related styrylquinazolinone analogues from 2-amino-*N*-phenylbenzamide, ethyl acetoacetate, and benzyl alcohol derivatives. In this case, optimization identified a dual DES system composed of DMU: L-(+)-tartaric acid and L-proline:PTSA, together with NaNO_3_, as the most effective combination, giving the model product in 87% yield at 90 °C after 3 h (Scheme 49). The method was further extended to a set of 16 styrylquinazolinones, and the substrate scope showed that electron-donating substituents were generally well tolerated, whereas *ortho*-substitution on the *N*-phenyl ring diminished the yields because of steric hindrance. Unlike the more straightforward benzoxazinone-based cyclizations discussed above, this work shows that DES-mediated quinazolinone chemistry can also support more elaborate tandem sequences leading to medicinally relevant targets [100].

**Scheme 50:**
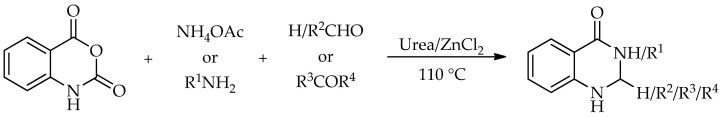
Multicomponent synthesis of 2,3-dihydroquinazolin-4(1*H*)-ones [101].

A DES-mediated multicomponent protocol based on isatoic anhydride, amines, and aldehydes or ketones was developed, with urea:ZnCl_2_ identified as the most effective eutectic mixture. Under optimized conditions, the model reaction yielded the target product in 97% after only 5 min at 110 °C. The method was extended to 18 dihydroquinazolin-4(1*H*)-ones, obtained in 92–98% yields. The same protocol was further applied to the synthesis of methaqualone, mecloqualone, and tryptanthrin, demonstrating that DES-mediated multicomponent strategies can also be useful for preparing biologically relevant targets (Scheme 50) [101].

**Scheme 51:**
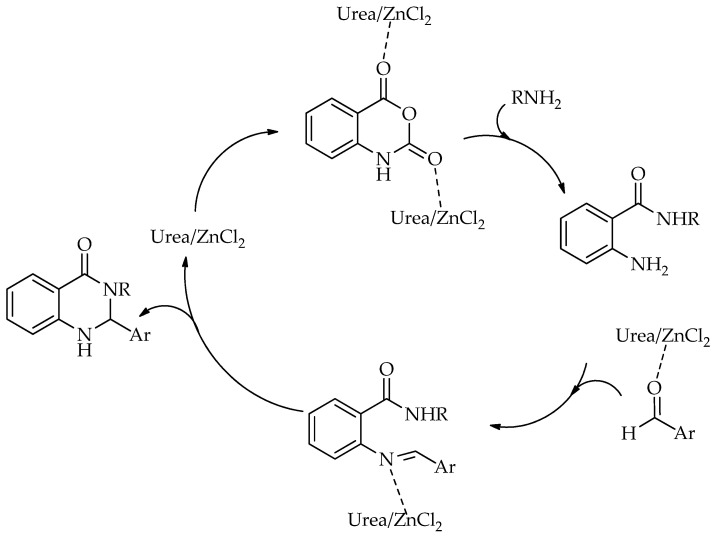
A plausible mechanism for the urea/ZnCl_2_-mediated synthesis of dihydroquinazolin-4(1*H*)-ones [101].

The formation of dihydroquinazolin-4(1*H*)-ones can be rationalized by the pathway shown in Scheme 51. The nucleophilic addition of the amine to the C4 carbonyl carbon of isatoic anhydride, facilitated by the DES, leads to ring opening, followed by decarboxylation to give anthranilamide as a key intermediate. The subsequent condensation of anthranilamide with the aldehyde affords a Schiff base, formed via the nucleophilic attack of the amino group on the activated carbonyl carbon. Intramolecular cyclization then occurs through the nucleophilic attack of the amide nitrogen on the azomethine carbon, leading to the dihydroquinazolinone framework. The catalytic role of the DES is attributed to ZnCl_2_ species, which likely enhance the electrophilicity of both carbonyl and imine functionalities, thereby facilitating the key transformation steps.

**Scheme 52:**
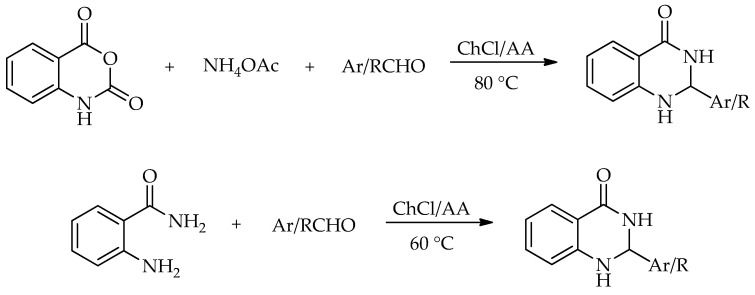
Synthesis of 2,3-dihydroquinazolin-4(1*H*)-ones in ChCl:AA [102].

**Scheme 53:**
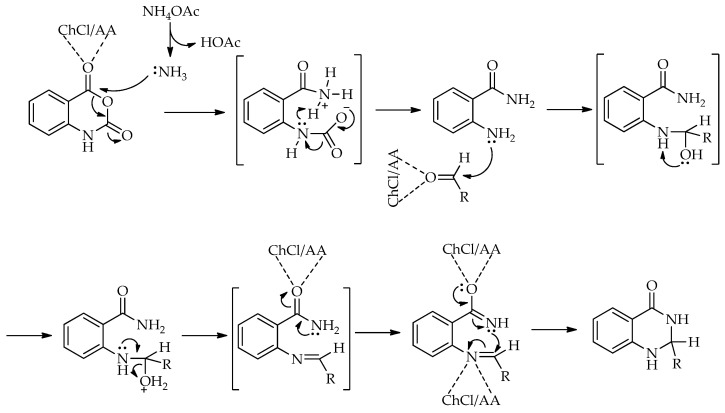
A plausible mechanism for the synthesis of 2,3-dihydroquinazoline-4(1*H*)-ones [102].

Hosseinzadeh et al. [102] employed a NADES composed of ChCl and ascorbic acid (AA) in the synthesis of dihydroquinazolin-4(1*H*)-ones (Scheme 52). In that study, two complementary protocols were developed in the same medium: the direct cyclocondensation of 2-aminobenzamide with aldehydes; and a three-component route from isatoic anhydride, aldehydes, and ammonium acetate. Optimization of the two-component model reaction showed that 110 mg of ChCl:AA at 60 °C gave the best result, affording the model product in 97% yield after only 10 min, whereas the three-component protocol was optimal at 80 °C and furnished the corresponding product in 93% yield after 30 min. Under these conditions, the direct route was extended to 18 dihydroquinazolin-4(1*H*)-ones in 76–97% yields, while the three-component variant provided nine examples in 76–94% yields (Scheme 53). The recovered NADES was reused for five cycles with only a moderate decline in activity [102].

The formation of 2,3-dihydroquinazolin-4(1*H*)-ones in the presence of ChCl/AA can be rationalized by the pathway shown in Scheme 53. Hydrogen-bonding interactions within the NADES activate the carbonyl group of isatoic anhydride, facilitating the nucleophilic attack of NH_4_OAc and subsequent ring opening followed by decarboxylation to give 2-aminobenzamide as a key intermediate. The condensation of this intermediate with the aldehyde affords an imine species after dehydration, with the carbonyl group activated through hydrogen bonding. The intramolecular nucleophilic attack of the amide nitrogen on the imine carbon then leads to cyclization, forming the dihydroquinazolinone. Final proton transfer steps yield the corresponding 2,3-dihydroquinazolin-4(1*H*)-one.

**Scheme 54:**
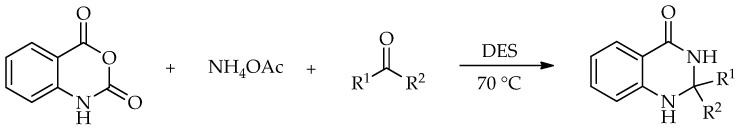
Synthesis of 2,3-dihydroquinazolin-4(1*H*)-ones in three-component DES [103].

Another recent example used a ternary NADES composed of ChCl, glycerol, and L-arginine as a dual solvent and catalyst system for the three-component synthesis of 2,3-dihydroquinazolin-4(1*H*)-ones from isatoic anhydride, ammonium acetate, and aldehydes or ketones (Scheme 54). Under the optimized conditions, only 0.2 mL of the medium was required to obtain the model product in 92% yield at 70 °C after 1.5 h. The method was applied to 17 derivatives (74–92%), and the recovered medium was reused three times without significant loss of efficiency.

**Scheme 55:**
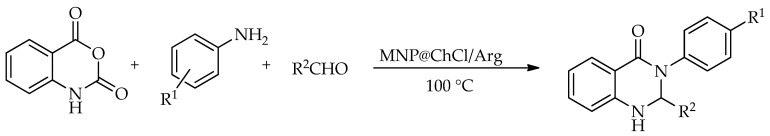
Synthesis of 2,3-dihydroquinazolin-4(1*H*)-ones by functionalized nanoparticles [103].

Valipour et al. introduced a heterogeneous DES-based catalyst for the preparation of 2,3-dihydroquinazolin-4(1*H*)-ones, in which a ChCl:arginine (Arg) system was immobilized on silica-coated magnetic nanoparticles and applied to the solvent-free three-component reaction of isatoic anhydride, aromatic amines, and aldehydes. Under optimized conditions, 30 mg of MNP@ChCl:Arg at 100 °C afforded the model product in 92% yield after 30 min, and the method was extended to 19 derivatives obtained in 85–93% yields (Scheme 55). The catalyst was easily recovered magnetically and reused for five cycles, demonstrating the practical advantage of transferring DES functionality onto a separable heterogeneous platform [103].

## 5. Conclusions

Deep eutectic solvents (DESs) have become important media in the synthesis of organic compounds, particularly heterocyclic compounds such as pyridine, coumarin and quinazolinone, which possess pharmaceutical significance. Their value lies in their dual or multifunctional role, as they can serve not only as environmentally friendly solvents, but also as catalysts and, in some cases, as reagents, further simplifying synthetic procedures and reducing the need for additional chemicals. In this context, DESs have proven to be a highly promising alternative to conventional organic solvents. Their main advantages include low toxicity, ease of preparation, frequent biodegradability, recyclability, and adaptability of physicochemical properties. In numerous examples, high yields, shorter reaction times, milder conditions, and simpler product isolation have been achieved, all of which contribute to reducing the environmental footprint of chemical processes. It is particularly noteworthy that the use of DESs enables efficient implementation of multicomponent reactions, “one-pot” synthesis, and combination with advanced techniques such as microwave heating, ultrasound and mechanochemistry. These approaches further increase the energy efficiency and selectivity of the process, in line with the principles of green chemistry. The strong hydrogen-bonding network within DESs enhances substrate activation and intermediate stabilization, contributing to improved selectivity and cleaner reaction profiles. Their application in the synthesis of biologically active heterocycles highlights not only improved chemical performance but also a clear advantage in reducing environmental impact. Consequently, DES-based methodologies represent a key advance in aligning synthetic organic chemistry with the principles of green chemistry.

However, despite numerous advantages, it is important to note that the “greenness” of DES systems is not universal and requires detailed assessment of the toxicity, biodegradability, and life cycle of individual systems. Therefore, further research should focus on standardizing the criteria for assessing the sustainability of DESs, as well as on the development of new, fully biocompatible and renewable systems (NADESs). Future research directions should include a deeper understanding of reaction mechanisms in DES media and the role of hydrogen bonds, broader application in electrochemical synthesis, and evaluation of economic feasibility under industrial conditions.

Overall, DESs represent a powerful tool for transforming traditional organic synthesis into a more sustainable and environmentally responsible process. Their application in the synthesis of biologically active heterocyclic compounds not only improves the efficiency of chemical reactions, but also actively contributes to the global goals of sustainable development and reducing the negative impact of the chemical industry on the environment.

## Data Availability

No new data were created or analyzed in this study.
